# Regioselective ring opening of aziridine for synthesizing azaheterocycle

**DOI:** 10.3389/fchem.2023.1280633

**Published:** 2023-10-19

**Authors:** Nikhil Srivastava, Hyun-Joon Ha

**Affiliations:** Department of Chemistry, Hankuk University of Foreign Studies, Yongin, Republic of Korea

**Keywords:** aziridine, regioselectivity, ring opening, pyrrolidine, piperidine

## Abstract

Aziridine had different regioselective ring openings depending on the functional group of its alkyl substituent. In the case of the alkyl group bearing γ-ketone at the C2 substituent of aziridine, the ring opening by the hydroxy nucleophile from H_2_O occurred by attacking the aziridine carbon at the C2 position. This reaction proceeded efficiently in the presence of CF_3_CO_2_H. Interestingly, the same starting aziridine ring bearing the alkyl substituent at the C2 position with the γ-silylated hydroxy group instead of γ-ketone led to the ring-opening reaction by the same oxygen nucleophile at the unsubstituted C3 position, with the breakage of the bond between aziridine N1 nitrogen and carbon at C3. These reaction products were cyclized to afford substituted pyrrolidine and piperidine rings with representative examples of congeners of pseudoconhydrine and monomorine.

## Graphical Abstract

**Figure:**
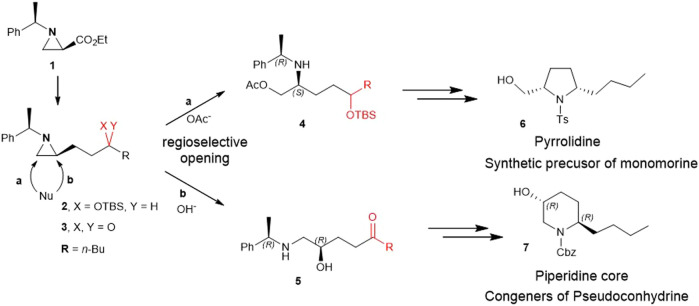
Regioselective ring opening of aziridine for synthesizing azaheterocycle.

## Introduction

**SCHEME 1:**
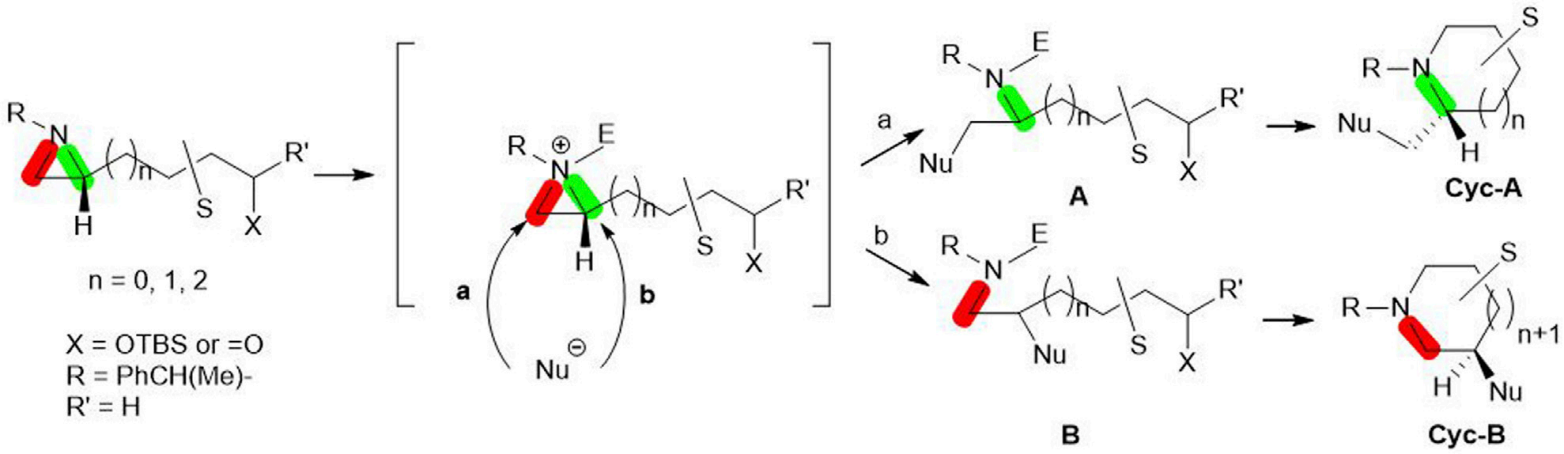
Aziridine ring-opening reactions by nucleophiles (either **a** or **b)** with breakage of bond C3-N (red) or C2-N (Green) to yield **A** or **B**. Subsequent cyclization of **A** or **B** decorated with the various functional groups(S) gives rise to either **Cyc-A** or **Cyc-B**.

Aziridine, a nitrogen-containing three-membered ring, has been used for the synthesis of various azaheterocycles based on its chemical reactivity and unique regio- and stereoselectivity. In our lab, we have synthesized various azaheterocycles utilizing chiral aziridine in its optically pure forms (Ha et al., 2014; D’hooghe and Ha, 2016). Biologically active compounds including alkaloids with azaheterocycles have been prepared from enantiopure aziridines via aziridine ring formation from its acyclic compounds or its ring transformation (Srivastava et al., 2020). Transformation is mostly based on the formation of aziridinium ion with proper electrophiles and the subsequent ring-opening by nucleophiles either at C2 (pathway **b** in Scheme 1) or C3 (pathway **a** in Scheme 1) (Dolfen et al., 2016; Choi et al., 2017; Ranjith and Ha, 2021; Ranjith and Ha, 2022). The nucleophilic ring opening at C2 or C3 is controlled by substituents at the aziridine ring, electrophiles, and nucleophiles to provide acyclic amine **A** or **B**. The substituent cyclization gives rise to **Cyc-A** or **Cyc-B** (Scheme 1) (Eum et al., 2015; Yadav et al., 2016; Srivastava et al., 2020).

**SCHEME 2:**
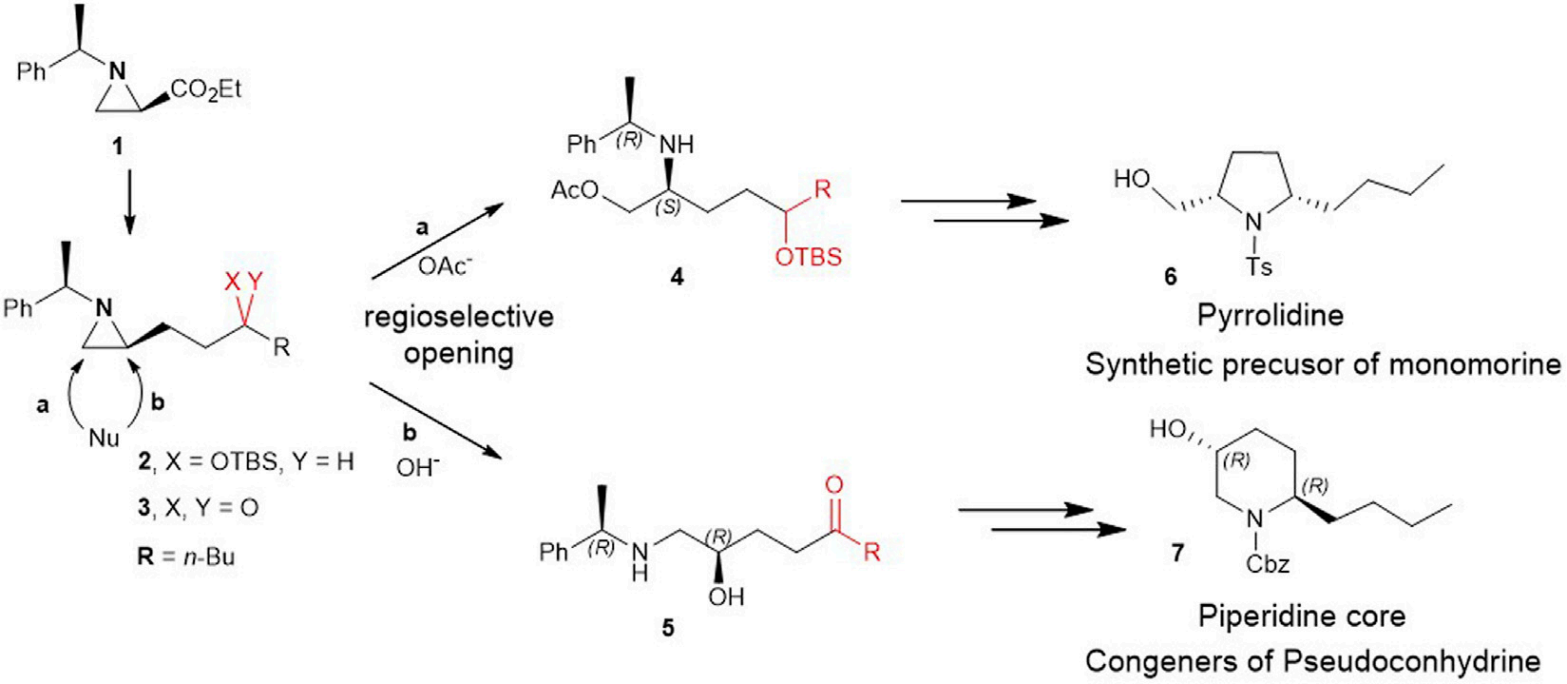
Ring-opening of 2-(3-hydroxy and 3-keto alkyl)aziridine (**2** and **3**) prepared from the same chiral aziridine-2-carboxylate (**1**) proceeds in a regioselective manner through either pathway **a** or **b** to yield **4** or **5** which is then cyclized to give pyrrolidine (**6**) or piperidine (**7**).

Herein, for the first time, we report the involvement of functionalization of 2-substituted aziridine-2-carboxylate to give piperidine alkaloids in an efficient regiochemical pathway of ring-opening reaction. More specifically, 2-(3 silylated-hydroxy and 3-keto alkyl) aziridine (**2** and **3**) prepared from the same chiral aziridine-2-carboxylate (**1**) reacted with nucleophiles for the ring-opening. These reactions proceeded in a regioselective manner through either pathway a or b to yield **4** or **5** which was then cyclized to give pyrrolidine (**6**) or piperidine (**7**) (Scheme 2).

## Results and discussion

At first, the ring opening of aziridine was performed to give rise to either terminal amine or internal amines with the breakage of bonds N1-C2 or N1-C3, respectively. For the preparation of piperidine ring, screening of ring-opening reactions were carried out with the compound 1-((*S*)-1-((*R*)-1-phenylethyl)aziridin-2-yl)oct-7-en-3-one (**3a**) as a model substrate in Table 1, which was derived from (2*R*)-aziridine-2-carboxylates (**1**) (Its synthetic procedure is described in experimental section).

**TABLE 1:** Screening of regioselective opening of ((*R*)-1-phenylethyl)aziridin-2-yl)oct-7-en-3-one (**3a**).

Entry	Protic acid^b^	Solvent	t [h]^c^	Yield of 8 (%)^d^
1	AcOH	Neat	4	No rxn
2	AcOH	1,4-Dioxane	5	No rxn
3	AcOH	Toluene	5	No rxn
4	AcOH	1,2-Dichloromethane	5	<8^e^
5	AcOH	1,2-Dichloroethane	6	No rxn
6	AcOH	THF	6	No rxn
7	TFA	CH_3_CN/H_2_O (9:1)	5	60
8	TFA	CH_3_CN/H_2_O (2:1**)**	5	75
9	TFA	Acetone/H_2_O(9:1)	4	80
10	**TFA**	**Acetone/H**_**2**_ **O (2:1)**	**4**	**90**
11	H_2_SO_4_	Acetone/H_2_O (2:1)	4	82

^a^ Reaction performed at 0.6 mmol of compound A.

^b^ Protic acid (1.0 equiv.).

^c^ Time in hours.

^d^ Isolated yield.

^e^ Acylated product 8.

Starting with a typical protocol for the ring openings of aziridines with neat acetic acid for 4 h at room temperature over the compound ((*R*)-1-phenylethyl)aziridin-2-yl)oct-7-en-3-one **3a**, did not afford any ring-opening product (Table 1, entry 1). Up to date, almost all regiochemical pathway takes to yield internal amines regardless of substituents at the side chain functionality including simple halogen, amine, and hydroxyl groups (Stankovic et al., 2012; Ha et al., 2014). Therefore, we expected internal amine as a ring-opening product with breakage of the bond between N1 and C3 without any substituent. After varying solvents such as 1,4-dioxane and toluene and performing the reaction with acetic acid (1 equiv) at room temperature for 5 h, no ring-opened product was obtained (Table 1, entries 2 and 3) with all starting material stayed as they were. We then switched to another solvent, dichloromethane. A regioselective product with OAc group (*R*)-5-oxo-1-(((*R*)-1-phenylethyl)amino)dec-9-en-2-yl acetate **8** was obtained in less than 10% yield (Table 1, entry 4). To increase the yield, the experiment was further carried out with the same acetic acid (1 equiv) in 1,2-dichloroethane and THF for 6 h. However, reactions ended up with the recovery of starting material only (Table 1, entries 5 and 6). Then we switched over to trifluoracetic acid (TFA) as another protic acid. When the representative substrate ((*R*)-1-phenylmethyl)aziridine-2-yl)oct-7-en-3-one **3a** was treated with TFA (1 equiv) in CH_3_CN:H_2_O (9:1) for 5 h at the same room temperature, unexpectedly, only regioselective product (*R*)-2-hydroxy-1-(((*R*)-1-phenylmethyl)amino)dec-9-en-5-one **8** was obtained in 60% yield (Table 1, entry 7). Changing the mixed solvent ratio of CH_3_CN:H_2_O from 9:1 to 2:1, the yield for compound **8** was improved to 75% under the same condition with TFA (1 equiv) (Table 1, entry 8). Inspired by this outcome, the starting compound **3a** was treated with the TFA (1 equiv) at room temperature under acetone instead of CH_3_CN. The yield was further improved to 80%. All these observations can be explained by the solubility and the way of the association between TFA and aziridine. This was further justified by changing the ratio of the acetone and H_2_O solvent system with the ratio from 9:1 to 2:1 to give its ring-opened product (*R*)-2-hydroxy-1-(((*R*)-1-phenylmethyl)amino)dec-9-en-5-one (**8**) as a single isomer in a 90% yield (Table 1, entry 10). In continuation, switching to strong bronsted acid i,e. sulfuric acid (H_2_SO_4_) (1N) (1 equiv) also gave efficient regioselective ring opened product **8** in 82% yield under same acetone and H_2_O (2:1) solvent system (Table 1, entry 11).

**FIGURE 1:**
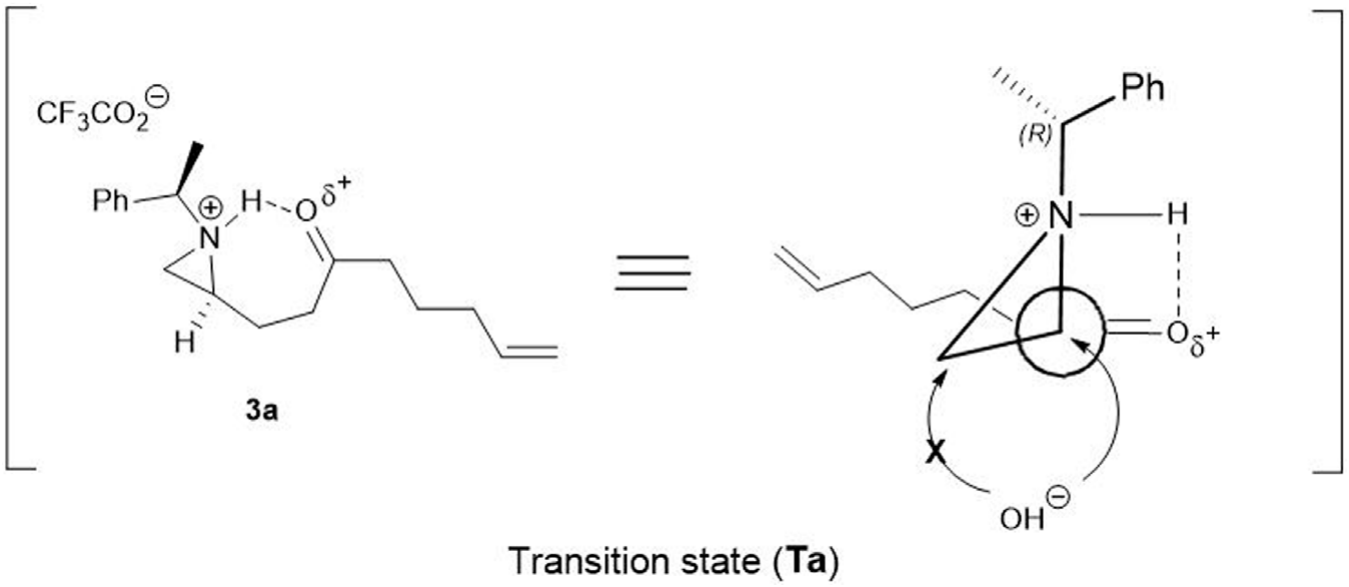
A schematic representation of plausible transition state (**Ta**) with keto compound **3a** for the regioselective opening of aziridine moiety.

On the basis of the above experiments described in Table 1, a plausible mechanism is proposed as shown in Figure 1. Possibly the reaction is mediated by the protonation of aziridine-nitrogen to form *in situ* aziridinium ion via a transition state (**Ta**). In this transition state, nitrogen attached proton, simultaneously interacted with oxygen attached to carbonyl carbon-oxygen *via* hydrogen bonding. Due to this transition state (**Ta**), the hydroxy nucleophile from H_2_O selectively approaches to attack at C2 carbon of the aziridinium ring exclusively in a regioselective manner (Figure 1) (Lopez and Salazar, 2013; Dalabehera et al., 2020). Under the same reaction conditions, other aziridinyl ketones such as 2-β- or 2-δ-ketoalkyl substituted aziridine yielded the mixture of the regioisomers of the ring-opened products which means that the conformation with fluorine is a driving force to determine the position of the nucleophilic attacks.

In order to support the plausible mechanistic pathway proposed above, we investigated ^1^H-NMR spectral change studies for the regioselective ring opening reaction over compound **3a** with TFA under deuterated acetone (d_6_)/D_2_O (2:1) solvent system (See in Supplementary Material S2; Figure 1). It was interesting to observe firstly an increase in δ ppm values in both N- benzylic (quartet at 4.00 ppm) and methyl protons (doublet at 1.85 ppm) after 20 min. It happened due to the formation of *in situ* N-aziridinium ion via transition state (**Ta**). Then after 1 h, the formation of a new quartet (at 4.63 ppm) of N-benzylic and a doublet of newly methyl protons at 1.81 ppm were observed, which confirmed the regioselective ring opening reaction process happened in a concerted manner. Finally, after 4 h formation of product **8** in a protonated form, via the regioselective ring opened process was fully confirmed during ^1^H-NMR change studies (See in Supplementary Material S2, Figure 1).

**SCHEME 3:**
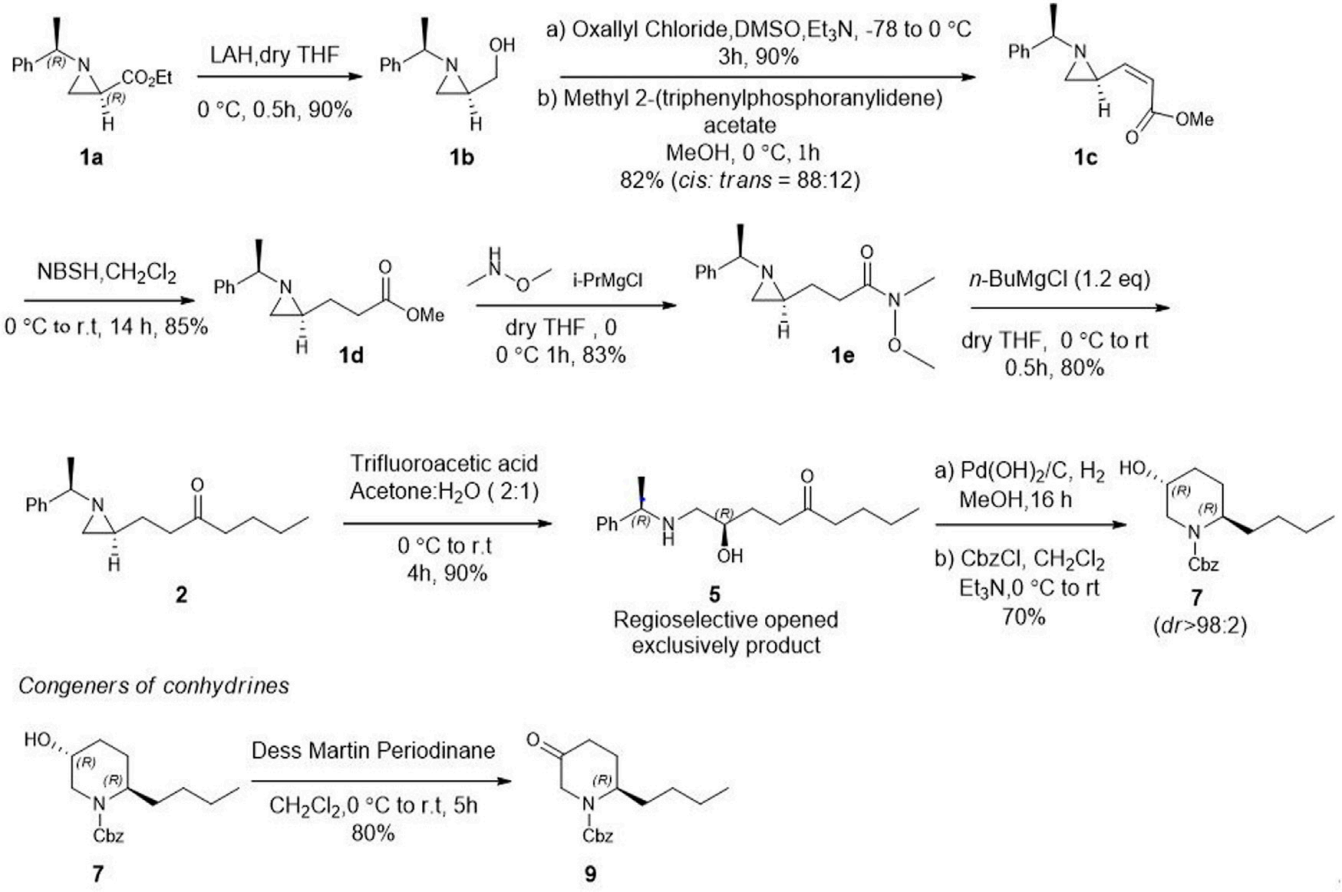
Asymmetric synthesis of congeners of pseudoconhydrine from (2*R*)-aziridine-2-carboxylate (**1**) via regioselective aziridine ring opening reaction as a key step.

**FIGURE 2:**
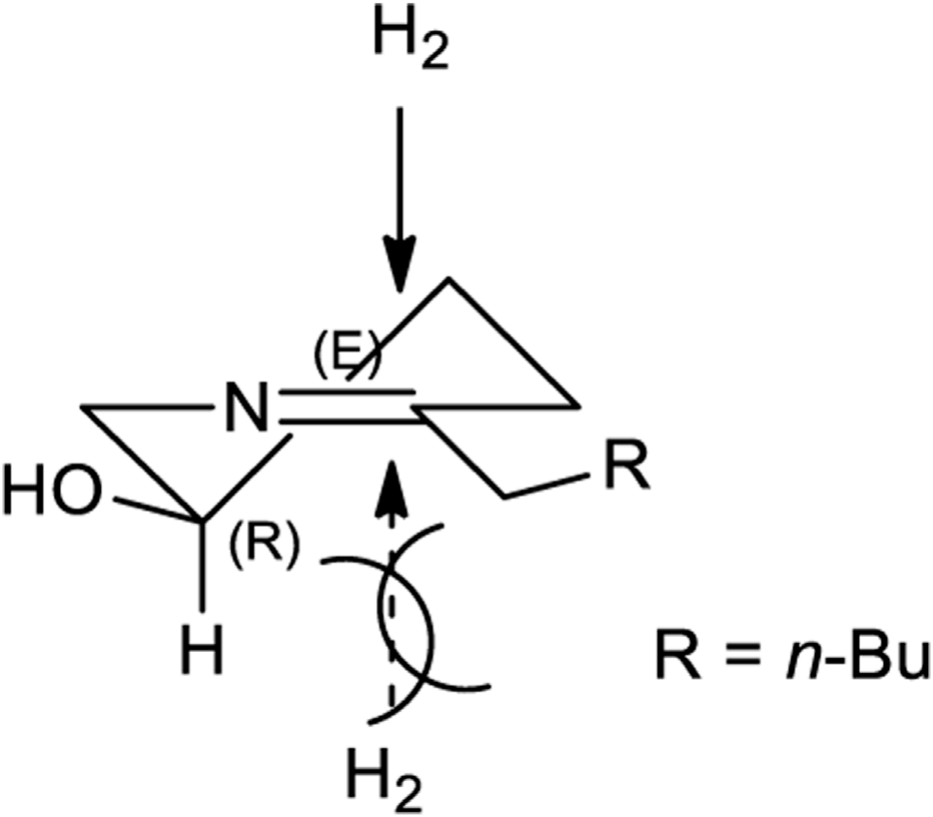
A Stereochemical aspect for one-pot hydrogenation of reductive amination for the synthesis of 2,5-*trans* piperidine core (**7**).

After establishing standard reaction conditions for regioselective aziridine ring opening, we then utilized this method for the synthesis of piperidine core **7** bearing substituents at C2. Those structures are cores of congeners analog to pseudoconhydrine (Scheme 3). Accordingly, we started our synthesis from chiral (2*R*)-aziridine-2-carboxylates (**1a**) as a starting material. The preparation of compound (*E*)-methyl 3-((*S*)-1-((*R*)-1-phenylethyl)aziridin-2-yl)acrylate (**1c**) was easily achieved from Swern oxidation of ((*R*)-1-((*R*)-1-phenylethyl)aziridin-2-yl)methanol (**1b**), followed by a Wittig reaction for the two carbon extension, resulting compound **1c** in a yield of 82% of isomers with the *cis:trans* ratio 88:12 using protocols for previously known sequential reactions (Scheme 3) (Lee and Ha, 2003; Yoon et al., 2010). After treatment with 2-nitrobenzenesulfonylhydrazide (NBSH), this olefinic product **1c** was then saturated to afford compound **1d** in 95% yield (Lee et al., 2009). The saturated methyl ester **1d** was then converted to Weinreb amide **1e**, followed by a reaction with *n*-C_4_H_9_MgCl, which afforded γ-aziridinyl ketone **2** in 80% yield (Macha and Ha, 2019). Based on the outcomes shown in Table 1, we then treated compound **2** following our established protocol for the regioselective aziridine ring-opening reaction with TFA under the mixed solvent system, acetone and H_2_O (2:1), at room temperature for 4 h. This yielded a hydroxy opened product **5** in 90% yield. Compound (*R*)-2-hydroxy-1-(((*R*)-1-phenylethyl)amino)nonan-5-one (**5**) was then treated with atmospheric H_2_ at room temperature under the catalytic amount of Pd(OH)_2_, yielding cyclic compound, followed by the reaction with CbzCl. These sequential reactions afforded *N*-Cbz-protected (2*S*,5*R*)-benzyl 2-butyl-5-hydroxypiperidine-1-carboxylate (**7**) (*dr* > 98:2) in 80% yield of two steps. The formation of compound **7** as a piperidine core skeleton (Macha et al., 2019) was obtained as a congener of pseudoconhydrine piperidine alkaloid (Bates et al., 2011). The formation of piperidine core **7** was executed with one-pot sequential debenzylation under hydrogenolysis followed by *in situ* cyclized via intramolecular reductive amination. Diastereoselectivity and assigned stereochemistry at the newly created center were then accessed. It was found that hydrogenation of a more stable intermediate imine could derive from a less hindered *β*-face of the molecule, resulting in a 2,5-*trans*-piperidine (Lee et al., 2003; Rao and Kumar, 2006) (Figure 2).

In addition, when compound **7** was further treated for oxidation under Dess-Martin periodinane, it produced 2-butyl-5-oxopiperidine (**9**) in 80% yield. Synthesizing this structural type of piperidone **9** (Ying et al., 2018; Lin et al., 2022) is an important intermediate to preparing various other biologically important 3-substituted piperidines (Vataku et al., 2014). The synthesis of 2-alkyl 5-hydroxy piperidine (**7**) and 2- alkyl 5-piperidone (**9**) with diverse substituents at C2 can be achieved efficiently using our regioselective opening protocol for γ-aziridinyl ketone which is prepared from commercially available (2*R*) aziridine −2 carboxylate at ease.

**SCHEME 4:**
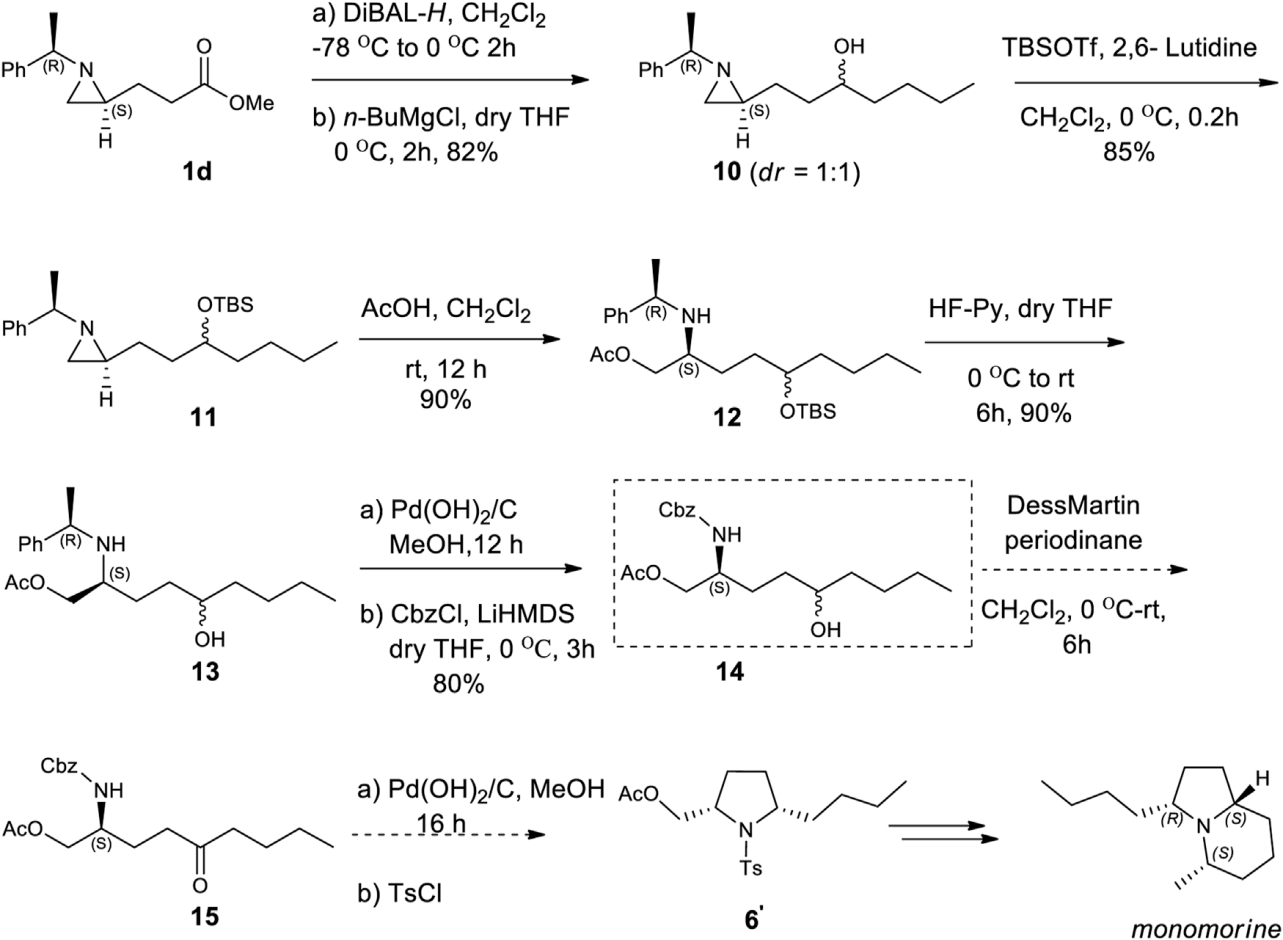
Asymmetric synthesis of monomorine from (2*R*)-aziridine-2-carboxylate (**1**) via regioselective aziridine ring opening reaction as a key step.

**FIGURE 3:**
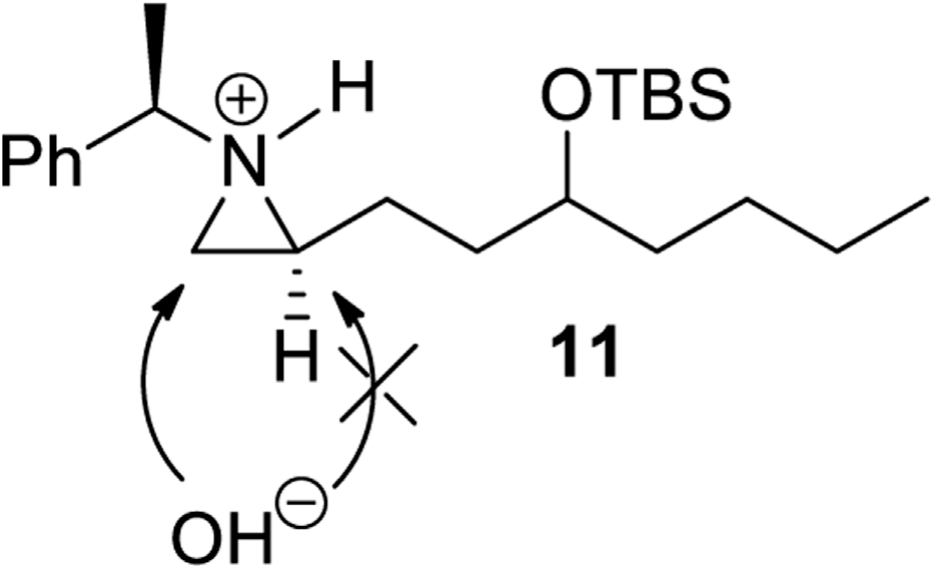
A plausible transition state for a regioselective opening of **11**.

Encouraged by the synthesis of piperidine core, we decided to synthesize pyrrolidine core moiety **11** in which two alkyl groups at C2 and C5 of pyrrolidine are in a *cis*-stereo relationship (Scheme 4). Accordingly, a regioselective aziridine ring opening driven by the breakage of the bond between N1 and C3 of 2-alkyl aziridine is needed. This could be again prepared from the same starting substrate **1d**, which was achieved from chiral aziridine-2-carboxylate (**1a**) at ease. Ester **1d** was reduced to aldehyde by DIBAL and then alkylated with *n-*butyl magnesium chloride in dry THF to give alcohol (**10**) in 82% yield for both diastereomers. The stereoselectivity of this alcohol was almost 1:1, which was not important because it would be oxidized for the formation of a pyrrolidine ring (Scheme 4). Both diastereomeric products **10** were treated with TBSOTf under 2,6-lutidine to afford silyl-protected **11** with a yield of 85%. Compound **11** was then subject to a regioselective aziridine ring-opening reaction under acetic acid conditions to give acetate-opened product **12** in a 90% yield (Singh et al., 2011). The drastic difference in the regioselectivity of **11** from the case of γ-ketoalkyl substituent (**2**) could be explained by the difference in the transition state (Figures 1, 3). A plausible transition state for the regioselective opening of **11** is only protonation of aziridine ring to form aziridinium ion in transition state **11**, followed by opening of aziridinium ion by oxygen nucleophiles from less-hindered C3 site (Figure 3).

As expected from the previous studies, we observed a ring-opened product with breakage of N1 and C3 bearing substituent which was different from a ring-opened product of γ-keto aziridine. To determine the regiochemical difference by acid, we treated compound **11** with CF_3_CO_2_H as shown in entry 10 of Table 1. It failed to give a ring-opened product with a decent yield. After achieving acetate compound **12**, it was treated under HF^.^ Pyridine condition in dry THF to give hydroxy compound **13** in a yield of 80%. Surprisingly, the triers to oxidize **13** under various oxidation conditions were unable to yield an oxidized product in a decent yield. Changing the 2-phenylethyl to N-Cbz as protecting group from sequential reactions consisting of debenzylation followed by CbzCl in LiHMDS base at 0 °C afforded compound **14** in 80% yield. With the known established protocol of oxidation (Srivastava and Ha, 2022) followed by deprotection of Cbz and cyclization under hydrogen in catalytic Pd(OH)_2_ gave 2,5-*cis* pyrrolidine (**6′**), the essential core skeleton of many pyrrolidines and indolizidine alkaloids including monomorine (Wang et al., 2009; Michael, 2016).

## Conclusion

In conclusion, we have successfully developed a regioselective ring opening reaction of aziridine moiety. The key feature includes the involvement of the functional group (γ-ketone or γ-silylated hydroxy group) present at the alkyl substituent of aziridine, which played a crucial role in the regioselective ring opening reaction of aziridine. In the case of γ-ketone group, the ring opening of aziridine occurred at carbon C2 position by hydroxy nucleophile from H_2_O under TFA condition in an efficient manner. Interestingly, in the case of γ-silylated hydroxy group present at the alkyl substituent of aziridine, the regioselective ring-opening reaction occurred at unsubstituted carbon C3 position of aziridine by the acetate oxygen nucleophile under acetic acid condition. These reaction products were cyclized to afford substituted pyrrolidine and piperidine rings with representative examples of congeners of pseudoconhydrine and monomorine.

## Experimental section

### Materials and methods

General Remarks. Chiral aziridine-2-carboxylates were obtained as their methyl ester from Sigma-Aldrich as reagents and from Imagene Co., Ltd. (http://www.imagene.co.kr/) in bulk quantities. Their corresponding ethyl esters were also obtained either from the transesterification of methyl ester or from Imagene (http://www.imagene.co.kr/) in bulk quantities. All commercially available compounds were used as received unless stated otherwise. All reactions were carried out under an atmosphere of nitrogen in oven-dried glassware with a magnetic stirrer. Reactions were monitored by thin layer chromatography (TLC) with 0.25 mm·E. Merck pre-coated silica gel plates (60 F254). Visualization was accomplished with either UV light or by immersion in a solution of ninhydrin, p-anisaldehyde, or phosphomolybdic acid (PMA) followed by heating on a hot plate for about 10 s. Purification of the reaction product was carried out by flash chromatography using Kieselgel 60 Art 9385 (230–400 mesh). 1H NMR and 13C NMR spectra were obtained using Varian unity INOVA 400WB (400 MHz) or Bruker AVANCE III HD (400 MHz) spectrometer. Chemical shifts are reported relative to chloroform (δ = 7.26) for 1H NMR and chloroform (δ = 77.0) for 13C{1H} proton-decoupled carbon NMR. Data are reported as (br = broad, s = singlet, d = doublet, t = triplet, q = quartet, p = quintet, m = multiplet). Coupling constants are given in Hz. Ambiguous assignments were resolved based on standard one-dimensional proton decoupling experiments. Optical rotations were obtained using a Rudolph Autopol III digital polarimeter and a JASCO P-2000. Optical rotation data are reported as follows [α]20 (concentration c = g/100 mL, solvent). High-resolution mass spectra were recorded on a 4.7 T Ion Spec ESI-TOFMS, JEOL (JMS-700). An AB Sciex 4800 Plus MALDI TOFTM (2,5-dihydroxybenzoic acid (DHB) matrix was used to prepare samples for MS. Data were obtained in the reflector positive mode with internal standards for calibration.

## Typical procedure and characteristic data

### 1-((S)-1-((R)-1-phenylethyl)aziridin-2-yl)oct-7-en-3-one compound: (3a)

To a stirred solution of chiral N-methoxy-N-methyl-3-((*S*)-1-((*R*)-1-phenylethyl)aziridin-2-yl)propanamide (1e) (500 mg, 1.9 mmol, 1.0 equiv.) in dry THF (25 mL) at 0°C, 4-pentenylmagnesium bromide solution C_5_H_9_MgBr (4.0 mL, 0.5 M in THF, 3.81 mmol) was added. The reaction mixture was slowly warmed to room temperature and stirred for 0.5 h. After completion as per TLC indication, the reaction was quenched with saturated NH_4_Cl (3 mL). The crude mixture was extracted with EtOAc (3 × 15 mL), dried over anhydrous Na_2_SO_4_, and concentrated under vacuum to afford crude product 3a, which was then purified by column chromatography to give compound 1-((*S*)-1-((*R*)-1-phenylethyl)aziridin-2-yl)oct-7-en-3-one (**3a**) as a viscous liquid (415 mg, 80% yield), TLC *R*_*f*_ (30% EtOAc/hexane = 0.30).

**[*α*]** ^**20**^_**D**_ = + 25.8 (*c* = 0.7, MeOH); ^**1**^ **H NMR** (400 MHz, CDCl_3_): *δ* 7.40–7.25 (m, 5 H), 5.87–5.73 (m, 1 H), 5.08–4.97 (m, 2 H), 2.68–2.57 (m, 2 H), 2.48 (t, *J* = 7.4 Hz, 2 H), 2.41 (q, *J* = 6.2 Hz, 1 H), 2.13–2.04 (m, 1 H), 2.02–1.89 (m, 1 H), 1.80–1.66 (m, 2 H), 1.60–1.53 (m, 2 H), 1.51–1.48 (m, 1 H), 1.43 (d, *J* = 6.6 Hz, 3 H), 1.32–1.27 (m, 1 H); ^**13**^ **C NMR** (101 MHz, CDCl_3_): 210.43, 144.44, 137.95, 128.24, 126.90, 126.73, 115.20, 69.60, 41.83, 40.48, 39.32, 33.47, 33.06, 27.05, 23.31, 22.70; **HRMS-ESI** (*m/z*) [M+H]^+^ calcd. for C_18_H_26_NO^+^ 272.2014; found 272.2006.

### *(R)-2-hydroxy-1-(((R)-1-phenylethyl)amino)dec-9-en-5-one:* (8)

To a stirred solution of 1-((*S*)-1-((*R*)-1-phenylethyl)aziridin-2-yl)oct-7-en-3-one (3a) (165 mg, 0.60 mmol) in acetone (2.0 mL) and water (1.0 mL) solvent system, trifluoro acetic acid (TFA) (0.05 mL, 0.87 mmol, 1.0 equiv.) was added at 0°C. The mixture was then stirred at room temperature for 4 h. After completion of the reaction per TLC indication, the reaction was quenched with a saturated solution of NaHCO_3_ (1 mL) and extracted with CH_2_Cl_2_ (4 × 10 mL). After concentrating under vacuum, the crude product was purified by flash column chromatography on silica gel to afford a pure product **8** (157 mg, 90%) TLC *R*_*f*_ (90% EtOAc/hexane = 0.1).

**[α]** ^**20**^_**D**_ = + 18.2 (*c* = 0.14, MeOH); ^**1**^ **H NMR** (400 MHz, CDCl_3_): *δ* 7.40–7.20 (m, 5 H), 5.86–5.68 (m, 1 H), 5.06–4.91 (m, 2 H), 3.81–3.70 (m, 1 H), 3.53–3.37 (m, 1 H), 2.63–2.45 (m, 2 H), 2.45–2.36 (m, 2 H), 2.11–1.96 (m, 2 H), 1.74–1.51 (m, 3 H), 1.42–1.33 (m, 3 H); ^**13**^ **C NMR** (101 MHz, CDCl_3_): *δ* 211.26, 144.82, 137.94, 128.55, 127.15, 126.45, 115.18, 69.36, 58.61, 53.38, 41.97, 38.91, 33.04, 28.55, 23.83, 22.77; **HRMS-ESI** (m/z) [M+H]^+^calcd. for C_18_H_28_NO_2_^+^ 290.2120; found 290.2115.

### *(Z)-methyl 3-((S)-1-((R)-1-phenylethyl)aziridin-2-yl)acrylate:* (1c)

To a stirred solution of oxalyl chloride (3.62 mL, 42.31 mmol, 1.5 equiv) in CH_2_Cl_2_ (80 mL) at −78°C, dimethyl sulfoxide (6.01 mL, 84.63 mmol, 3.0 equiv.) was added over 15 min. The resulting mixture was stirred for another 40 min. Then a solution of ((*R*)-1-((*R*)-1-phenylethyl)aziridin-2-yl)methanol **1b** (5.0 g, 28.21 mmol) in CH_2_Cl_2_ (30 mL) was added dropwise at the same temperature. The reaction mixture was stirred for 1.5 h at the same temperature. Triethylamine (11.81 mL, 42.31 mmol, 3.0 equiv.) was then added at −78°C and allowed to stir at the same temperature for 30 min. It was then warmed to 0°C and stirred for 30 min. After completion of the reaction per TLC indication, the reaction mixture was quenched with water (80 mL) and extracted with CH_2_Cl_2_ (2 × 100 mL). Combined organic layers were dried over anhydrous Na_2_SO_4_ and concentrated under reduced pressure to obtain a crude aldehyde which was used for the Wittig reaction without further purification.

To a solution of [(1*R*)-phenylethylaziridine]-(2*R*)-carboxaldehyde (3.0 g, 17.12 mmol, 1.0 equiv) in MeOH (34 mL), methyl triphenylphosphoranylideneacetate (6.86 g, 20.54 mmol, 1.2 equiv.) was added at 0°C. The resulting mixture was allowed to stir for 1 h at 0°C. After completion of the reaction, methanol was removed under vacuum and H_2_O (2 × 50 mL) was added to the reaction mixture. The organic layer was then extracted with CH_2_Cl_2_ (3 × 100 mL), dried over anhydrous Na_2_SO_4_, and concentrated under a vacuum. The crude product was purified by flash column chromatography on silica gel to afford a pure product (Z)-methyl 3-((S)-1-((R)-1-phenylethyl) aziridin-2-yl)acrylate **1c** (3.2 g, 82%) TLC *R*_*f*_ (30% EtOAc/hexane = 0.4).

**[α]**_**D**_ = +58.3 (*c* = 16.20, in CHCl_3_); ^**1**^ **H NMR** (400 MHz, CDCl_3_): *δ* 7.43–7.22 (m, 6 H), 6.78 (dt, *J* = 13.1, 6.6 Hz, 1 H), 6.20–6.09 (m, 1 H), 5.98–5.82 (m, 2 H), 3.78 (s, 3 H), 2.66–2.59 (m, 1 H), 2.21–2.10 (m, 1 H), 1.86–1.72 (m, 2 H), 1.70–1.63 (m, 1 H), 1.47 (t, *J* = 6.8 Hz, 1 H), 1.43 (dd, *J* = 6.5, 1.6 Hz, 1 H); ^**13**^ **C NMR** (101 MHz, CDCl_3_): *δ* 166.57, 150.51, 148.54, 143.78, 128.25, 127.01, 126.64, 126.61, 121.22, 120.17, 69.62, 51.09, 39.45, 37.32, 22.89; **HRMS-ESI** (*m/z*) [M+1]^+^calcd. for C_14_H_18_NO_2_^+^ 231.1337; found C_14_H_18_NO_2_^+^- H+ 231.1331.

### *Methyl 3-((S)-1-((R)-1-phenylethyl)aziridin-2-yl)propanoate* (1d)

To a stirred solution of olefin, **1c** (2.0 g, 8.64 mmol) in CH_2_Cl_2_ (24 mL) at 0°C, 2-nitrobenzenesulfonylhydrazide (NBSH) (7.5 g, 34.58 mmol, 4.0 equiv) was added. Then triethylamine (10.0 mL, 69.17 mmol, 8.0 equiv) was added in a dropwise manner at the same 0°C. The reaction mixture was then allowed to stir at room temperature for an additional 12 h. After completion of starting material as confirmed by TLC, the reaction mixture was quenched with saturated NaHCO_3_ solution, extracted with CH_2_Cl_2_ (3 × 50 mL), and concentrated under vacuum to give a crude product 1d, which was then purified by column chromatography to give compound methyl 3-((*S*)-1-((*R*)-1-phenylethyl)aziridin-2-yl)propanoate (**1d**) as a viscous liquid (1.72 g, 85% yield), TLC *R*_*f*_ (40% EtOAc/hexane = 0.30).

**[*α*]** ^**20**^_**D**_ = + 35.4 (*c* = 0.32, MeOH); ^**1**^ **H NMR** (400 MHz, CDCl_3_): *δ* 7.40–7.23 (m, 5 H), 3.72 (s, 3 H), 2.58–2.51 (m, 2 H), 2.42 (q, *J* = 6.5 Hz, 1 H), 2.02–1.93 (m, 1 H), 1.65–1.58 (m, 2 H), 1.52 (d, *J* = 3.2 Hz, 1H), 1.44 (d, *J* = 6.6 Hz, 3 H), 1.36–1.27 (m, 1 H); ^**13**^ **C NMR** (101 MHz, CDCl_3_) *δ* 173.71, 144.49, 128.22, 126.87, 126.71, 69.60, 51.52, 39.10, 33.41, 31.91, 28.26, 23.27; **HRMS-ESI** (*m/z*) [M+H]^+^calcd. for C_14_H_20_NO_2_^+^ 234.1494; found 234.1486.

### *N-methoxy-N-methyl-3-((S)-1-((R)-1-phenylethyl)aziridin-2-yl)propanamide* (1e)

To a stirred solution of saturated ester **1d** (1.5 g, 6.42 mmol, 1.0 equiv.) and N,O-dimethylhydroxylamine hydrochloride (1.15 g, 9.64 mmol, 1.5 equiv.) in dry THF (22 mL) at 0°C, *i*-PrMgCl (9.6 mL, 2.0 M in THF, 19.28 mmol, 3.0 equiv.) was slowly added. The reaction mixture was stirred for 1 h at the same 0°C. After completion of the reaction as per TLC, the reaction mixture was quenched with saturated NH_4_Cl solution (2 mL) and extracted with EtOAc (4 × 50 mL). Combined organic layers were dried over anhydrous Na_2_SO_4_. Under vacuum, solvents were removed to obtain a crude amide product, which was purified by column chromatography to give a pure Weinreb amide product (**1e**) (1.40 g, 83% yield), TLC *R*_*f*_ (90% EtOAc/hexane = 0.1).

**[*α*]** ^**20**^_**D**_ = + 28.3 (*c* = 0.70, MeOH); ^**1**^ **H NMR** (400 MHz, CDCl3): *δ* 7.37–7.15 (m, 5 H), 3.63 (d, *J* = 14.7 Hz, 3 H), 3.17–3.09 (m, 3 H), 2.60 (dd, *J* = 14.3, 8.6 Hz, 2 H), 2.37 (q, *J* = 6.5 Hz, 1 H), 1.98–1.86 (m, 1 H), 1.65–1.51 (m, 2 H), 1.46 (d, *J* = 3.3 Hz, 1 H), 1.39 (d, *J* = 6.6 Hz, 3 H), 1.25 (d, *J* = 6.3 Hz, 1 H);^**13**^ **C NMR** (101 MHz, CDCl_3_): *δ* 173.76, 144.20, 127.90, 126.54, 126.43, 69.23, 60.81, 39.24, 33.25, 31.83, 29.36, 27.46, 23.01; **HRMS-ESI** (*m/z*) [M+H]^+^calcd. for C_15_H_23_N_2_O_2_^+^ 263.1759; found 263.1751.

### *1-((S)-1-((R)-1-phenylethyl)aziridin-2-yl)heptan-3-one:* (2)

To a stirred solution of Weinreb amide **1e** (1.0 g, 3.8 mmol, 1.0 equiv.) in dry THF (50 mL) at 0°C, *n*-Butylmagnesium chloride solution C_4_H_9_MgCl (1.9 mL, 2.0 M in Ether, 1.90 mmol) was added. The reaction mixture was slowly warmed to room temperature and stirred for 0.5 h. After completion of the reaction as per TLC, the reaction mixture was quenched with NH_4_Cl solution and extracted with EtOAc (3 × 80 mL). Combined organic layers were dried over Na_2_SO_4_ and concentrated *in vacuo*, which was then purified by silica gel column chromatography to afford ketone product **2** (830 mg, 80% yield) TLC *R*_*f*_ (60% EtOAc/hexane = 0.5).

**[*α*]** ^**20**^_**D**_ = + 51.0 (*c* = 0.06, MeOH); ^**1**^ **H NMR** (400 MHz, CDCl_3_): *δ* 7.42–7.32 (m, 3 H), 7.31–7.21 (m, 2 H), 2.70–2.58 (m, 1 H), 2.47 (dd, *J* = 9.3, 5.6 Hz, 2 H), 2.41 (q, *J* = 6.5 Hz, 1 H), 2.01–1.89 (m, 3 H), 1.66–1.53 (m, 1 H), 1.52–1.48 (m, 1 H), 1.43 (d, *J* = 6.6 Hz, 3 H), 1.40–1.26 (m, 1 H), 0.98–0.90 (m, 3 H); ^**13**^ **C NMR** (101 MHz, CDCl_3_): *δ* 210.77, 144.51, 128.26, 126.91, 126.75, 69.64, 42.52, 40.40, 39.36, 33.49, 27.10, 25.94, 23.32, 22.35, 13.85; **HRMS-ESI** (*m/z*) [M+H]^+^calcd. for C_17_H_26_NO^+^ 260.2014; found 260.2003.

### *(R)-2-hydroxy-1-(((R)-1-phenylethyl)amino)nonan-5-one:* (5)

The procedure was analogous to that used for preparing compound **8** using 1-((*S*)-1-((*R*)-1-phenylethyl)aziridin-2-yl)heptan-3-one (**2**) (800 mg, 3.084 mmol, 1.0 equiv.) in an acetone (10.0 mL) and water (5.0 mL) solvent system with trifluoro acetic acid (TFA) (0.23 mL, 3.08 mmol, 1.0 equiv) to afford compound (*R*)-2-hydroxy-1-(((*R*)-1-phenylethyl)amino)nonan-5-one (**5**) (760 mg, 90% yield), TLC *R*_*f*_ (90% EtOAc/hexane = 0.1).

**[*α*]** ^**20**^_**D**_ = + 87.2 (*c* = 0.14, MeOH); ^**1**^ **H NMR** (400 MHz, CDCl_3_): 7.49–7.11 (m, 5H), 3.87–3.66 (m, 1H), 3.54–3.32 (m, 1H), 2.63–2.43 (m, 3H), 2.40–2.31 (m, 3H), 1.77–1.63 (m, 1H), 1.61–1.46 (m, 3H), 1.44–1.36 (m, 3H), 1.32–1.20 (m, 2H), 0.96–0.82 (m, 3H); ^**13**^ **C NMR** (101 MHz, CDCl_3_): *δ* 211.63, 144.80, 128.57, 127.19, 126.48, 69.35, 58.61, 53.37, 42.64, 38.82, 28.56, 25.96, 23.79, 22.32, 13.83; **HRMS**-ESI (*m/z*) [M+H]^+^calcd. for C_17_H_28_NO_2_^+^ 278.2120; found 278.2114.

### *(2R,5R)-benzyl 2-butyl-5-hydroxypiperidine-1-carboxylate:* (7)

Compound **5** (600 mg, 2.16 mmol) was taken in MeOH (10 mL) and degassed with N_2_ for 2 h. Then 20% Pd(OH)_2_/C (242 mg, 1.7 mmol, 0.8 equiv) was added and the mixture was hydrogenated under an atmospheric pressure of hydrogen for 16 h. After completion of the reaction as per TLC, the reaction mixture was diluted with MeOH (20 mL) and filtered on a pad of Celite using MeOH as solvent. The filtrate was concentrated under vacuum and crude product was used for N-Cbz protection reaction without purification.

To a solution of crude product (325 mg) in dry CH_2_Cl_2_ (5 mL), triethyl amine (0.5 mL, 3.25 mmol, 1.6 equiv.) and benzyl chloroformate (CbzCl) (0.43 mL, 3.05 mmol, 1.5 equiv.) were added at 0°C. Then resulting mixture was allowed to stir at room temperature for 3 h. After completion of the reaction, the reaction was quenched with H_2_O (2 mL). The organic layer was then extracted with CH_2_Cl_2_ (2 × 20 mL), dried over anhydrous Na_2_SO_4_, and concentrated under vacuum to give an *N*-Cbz protected piperidine crude product, which was then purified by flash column chromatography on silica gel to afford a pure product (2*R*,5*R*)-benzyl 2-butyl-5-hydroxypiperidine-1-carboxylate (**7**) (412 mg, 70% yield for 2 steps) TLC *R*_*f*_ (40% EtOAc/hexane = 0.3).

**[α]** ^**20**^_**D**_ = −9.3 (*c* = 0.30, MeOH); ^**1**^ **H NMR** (400 MHz, CDCl_3_): *δ* 7.44–7.29 (m, 5H), 5.16 (q, *J* = 12.3 Hz, 2H), 4.33 (s, 1H), 4.12 (s, 1H), 3.90 (d, *J* = 38.5 Hz, 1H), 3.05 (d, *J* = 14.0 Hz, 1H), 2.21–1.93 (m, 2H), 1.84–1.58 (m, 3H), 1.46–1.11 (m, 6H), 0.88 (t, *J* = 6.4 Hz, 3H); ^**13**^ **C NMR** (101 MHz, CDCl_3_): *δ* 156.52, 136.76, 128.42, 127.89, 127.77, 67.08, 64.47, 50.59, 44.76, 28.73, 28.46, 25.40, 22.54, 14.05; **HRMS**-ESI (*m/z*) [M+H]^+^calcd. for C_17_H_26_NO_3_^+^ 292.1912; found 292.1905.

### (R)*-benzyl 2-butyl-5-oxopiperidine-1-carboxylate:* (9)

To a stirred solution of **7** (200 mg, 0.68 mmol, 1.0 equiv) in CH_2_Cl_2_ (15 mL), Dess-Martin periodinane (DMP) (581 mg, 1.37 mmol, 2.0 equiv) was added at 0°C. After 10 min, the reaction mixture was allowed to stir for an additional 8 h at room temperature. After completion of the reaction as per TLC, the reaction mixture was filtered on a pad of Celite using CH_2_Cl_2_ (2 × 5 mL) as solvent. The saturated NaHCO_3_ (5 mL) was used to treat the filtrate. Combined organic extracts were washed with CH_2_Cl_2_ (2 × 15 mL), dried over anhydrous Na_2_SO_4_, and concentrated *in vacuo*. The residue was purified by flash chromatography on silica gel to provide keto compound **9** (158 mg, 80%) as a liquid, TLC *R*_*f*_ (30% EtOAc/hexane = 0.5).

**[*α*]** ^**20**^_**D**_ **=** +48.0 (*c* = 0.55, MeOH); ^**1**^ **H NMR** (400 MHz, CDCl_3_): *δ* 7.50–7.32 (m, 5H), 5.28–5.08 (m, 2H), 4.68–4.44 (m, 1H), 4.40–4.18 (m, 1H), 3.62 (d, *J* = 17.7 Hz, 1H), 2.44 (t, *J* = 6.7 Hz, 2H), 2.24 (m, 1H), 1.68 (m, 2H), 1.54 (m, 1H), 1.32 (m, 4H), 0.98–0.84 (m, 3H); ^**13**^ **C NMR** (101 MHz, CDCl_3_ **)**: *δ* 207.37, 166.95, 136.29, 128.52, 128.16, 127.99, 67.50, 50.81, 50.40, 35.69, 27.90, 22.51, 13.97; **HRMS-ESI** (*m/z*) [M+H]^**+**^calcd. for C_17_H_24_NO_3_^+^ 290.1756; found 290.1748.

### *1-((S)-1-((R)-1-phenylethyl)aziridin-2-yl)heptan-3-ol:* (10)

To a stirred solution of methyl 3-((*S*)-1-((*R*)-1-phenylethyl) aziridine-2-yl)propanoate **(1d)** (2 g, 8.57 mmol, 1.0 equiv) in dry CH_2_Cl_2_ (40 mL), a solution of diisobutylaluminum hydride solution (DIBAL-*H*) (9.4 mL, 1.0 M in Toluene, 9.43 mmol) was added dropwise at—78°C. The reaction mixture was allowed to stir for 2 h at the same temperature. After completion of the reaction as per TLC indication, the reaction was quenched with a saturated solution of Na-k tartarate (10 mL) at 0°C. The reaction mixture was then stirred for an additional 1 h. The crude mixture was extracted with CH_2_Cl_2_ (3 × 20 mL), dried over anhydrous Na_2_SO_4_, and concentrated under vacuum to afford crude aldehyde, which was used for the next step without further purification.

To a stirred solution of crude aldehyde in dry THF (40 mL) at 0°C, *n*-butylmagnesium chloride solution C_4_H_9_MgCl (4.1 mL, 2.0 M in ether, 4.0 mmol) was added. The reaction mixture was allowed to stir for 2 h at the same temperature. After completion of the reaction per TLC indication, the reaction mixture was quenched with saturated NH_4_Cl solution and extracted with EtOAc (2 × 100 mL). Combined organic layers were dried over Na_2_SO_4_ and concentrated *in vacuo* to afford a crude product which was then purified by silica gel column chromatography to afford diastereomeric (1:1) hydroxy compound **10** (1.82 g, 80% yield for two steps) at the same *R*_*f*_, TLC *R*_*f*_ (40% EtOAc/hexane = 0.5).

**[*α*]** ^**20**^_**D**_ **=** + 55.2 (*c* = 0.11, MeOH);^**1**^ **H NMR** (400 MHz, CDCl_3_): *δ* 7.43–7.24 (m, 10H), 3.67–3.52 (m, 1H), 2.56–2.50 (m, 1H), 2.49–2.43 (m, 1H), 2.06–1.88 (m, 1H), 1.79–1.69 (m, 2H), 1.69–1.64 (m, 2H), 1.60 (qd, *J* = 4.0, 1.9 Hz, 3H), 1.54–1.42 (m, 11H), 1.41–1.31 (m, 4H), 1.30–1.26 (m, 1H), 1.04–0.85 (m, 6H); ^**13**^ **C NMR** (101 MHz, CDCl_3_): *δ* 144.22, 144.04, 128.31, 127.00, 126.75, 71.56, 69.81, 39.96, 39.68, 37.34, 33.88, 33.21, 28.82, 28.67, 27.97, 23.37, 22.84, 14.08; **HRMS**-**ESI** (*m/z*) [M+H]^**+**^calcd. for C_17_H_27_NO^+^ 262.2171; found 262.2160.

### *(2S)-2-(3-((tert-butyldimethylsilyl)oxy)heptyl)-1-((R)-1-phenylethyl)aziridine:* (11)

Tert-butyldimethylsilyltrifluoromethane sulfonate (TBSOTf) (1.5 mL, 6.73 mmol, 1.1 equiv) was slowly added to a cooled solution (0°C) of alcohol **10** (1.6 g, 6.12 mmol, 1.0 equiv) and 2,6-lutidine (1.4 mL, 12.24 mmol, 2.0 equiv) in dry CH_2_Cl_2_ (30 mL). After 20 min, the reaction mixture was diluted with CH_2_Cl_2_ (20 mL), quenched with water, and extracted with CH_2_Cl_2_ (2 × 15 mL). Combined organic layers were dried over anhydrous Na_2_SO_4_ and concentrated *in vacuo* to obtain a crude product which was purified by flash column chromatography to afford TBS protected compound **11** (1.92 g, 85% yield) TLC *R*_*f*_ (20% EtOAc/hexane = 0.6).

**[*α*]** ^**20**^_**D**_ = + 26.2 (*c* = 0.5, MeOH); ^**1**^ **H NMR** (400 MHz, CDCl_3_): *δ* 7.46–7.26 (m, 10H), 3.90–3.53 (m, 2H), 2.46–2.34 (m, 2H), 1.85–1.51 (m, 3H), 1.51–1.43 (m, 10H), 1.39–1.23 (m, 6H), 0.96–0.86 (m, 18H), 0.12–0.06 (m, 6H), 0.04 (s, 6H); ^**13**^ **C NMR** (101 MHz, CDCl_3_): *δ* 144.65, 128.24, 126.84, 72.27, 72.21, 71.92, 71.84, 69.89, 40.76, 37.12, 36.57, 35.14, 34.98, 33.36, 29.42, 28.64, 27.50, 25.95, 25.91, 25.70, 25.66, 23.29, 22.87, 18.14, 18.11, 14.11, −2.96, −4.37; **HRMS**-**ESI** (*m/z*) [M+H]^**+**^calcd. for C_23_H_42_NOSi^+^ 376.3035; found 376.3023.

### *(2S)-5-((tert-butyldimethylsilyl)oxy)-2-(((R)-1-phenylethyl)amino)nonyl acetate* (12)

To (2*S*)-2-(3-((*tert-*butyldimethylsilyl)oxy)heptyl)-1-((*R*)-1-phenylethyl)aziridine (**11**) (800 mg, 2.12 mmol) in CH_2_Cl_2_ (10 mL), acetic acid (0.3 mL, 4.25 mmol, 2.5 equiv) was added at 0°C. The reaction mixture was allowed to stir at room temperature for 12 h. After completion of the reaction per TLC indication, the reaction mixture was quenched with a saturated solution of NaHCO_3_ (5 mL), extracted with CH_2_Cl_2_ (3 × 15 mL), and concentrated under *vacuum* to obtain a crude product which was then purified by flash column chromatography on silica gel to afford a pure product **12** (820 mg, 89%) TLC *R*_*f*_ (20% EtOAc/hexane = 0.5).

**[*α*]** ^**20**^_**D**_ = + 38.2 (*c* = 0.70, MeOH);^**1**^ **H NMR** (400 MHz, CDCl_3_): *δ* 7.40–7.16 (m, 10H), 4.05–3.94 (m, 1H), 3.92–3.81 (m, 3H), 3.71–3.53 (m, 1H), 2.72–2.45 (m, 1H), 2.07–2.01 (m, 6H), 1.62–1.53 (m, 3H), 1.50–1.38 (m, 3H), 1.36–1.31 (m, 4H), 1.30–1.21 (m, 3H), 0.92–0.85 (m, 18H), 0.05–0.00 (m, 12H); ^**13**^ **C NMR** (101 MHz, CDCl_3_): *δ* 171.01, 145.76, 145.72, 128.40, 126.93, 126.52, 72.06, 72.02, 66.86, 66.83, 55.23, 55.17, 53.61, 36.62, 32.59, 27.49, 26.50, 25.91, 24.79, 22.86, 20.92, 18.10, 14.11, −4.47; **HRMS**-**ESI** (*m/z*) [M+H]^**+**^calcd. for C_25_H_46_NO_3_Si^+^ 436.3247; found 436.3234.

### *(2S)-5-hydroxy-2-(((R)-1-phenylethyl)amino)nonyl acetate* (13)

To a stirred solution of **12** (500 mg, 1.14 mmol) in dry THF (12 mL) in a polypropylene vial HF-Py complex (70%, 0.4 mL) at 0°C was added. The reaction mixture was slowly raised to room temperature and stirred for 12 h. After completion of the reaction per TLC indication, the reaction mixture was cautiously quenched with saturated aqueous NaHCO_3_ and stirred for 20 min. Then both layers were separated. The aqueous layer was further extracted with EtOAc (2 × 20 mL). Combined organic layers were washed with saturated aqueous CuSO_4_ (5 mL), water (5 mL), and brine (5 mL), dried over Na_2_SO_4_, and concentrated *in vacuo* to obtain a crude product, which was then purified by flash column chromatography on silica gel to afford a pure product **13** (295 mg, 80%) TLC *R*_*f*_ (70% EtOAc/hexane = 0.2).

**[*α*]** ^**20**^_**D**_ **=**—51.2 (*c* = 0.22, MeOH); ^**1**^ **H NMR** (400 MHz, CDCl_3_): *δ* 7.51–7.12 (m, 10H), 4.17–4.02 (m, 2H), 4.02–3.94 (m, 2H), 3.93–3.81 (m, 2H), 3.63–3.43 (m, 1H), 3.06–2.81 (m, 1H), 2.81–2.60 (m, 1H), 2.13–1.94 (m, 6H), 1.84–1.45 (m, 4H), 1.46–1.16 (m, 9H), 1.03–0.71 (m, 6H); ^**13**^ **C NMR** (101 MHz, CDCl_3_): *δ* 170.77, 170.67, 144.95, 144.65, 128.51, 128.47, 127.18, 127.12, 126.38, 126.26, 71.66, 71.25, 66.17, 65.82, 55.22, 54.92, 54.20, 52.68, 37.22, 37.15, 34.45, 32.82, 29.59, 27.95, 27.92, 27.59, 23.78, 23.00, 22.71, 22.69, 20.71, 13.99; **HRMS**-**ESI** (*m/z*) [M+H]^**+**^calcd. for C_19_H_32_NO_3_^+^ 322.2382; found 322.2372.

### *(2S)-2-(((benzyloxy)carbonyl)amino)-5-hydroxynonyl acetate:* (14)

Compound **13** (250 mg, 0.77 mmol) was taken in MeOH (10 mL) and degassed with N_2_ for 1 h. Then 20% Pd(OH)_2_/C (218 mg, 1.55 mmol, 2.0 equiv) was added and the mixture was hydrogenated under an atmospheric pressure of hydrogen for 16 h. The reaction mixture was diluted with MeOH (15 mL) and filtered on a pad of Celite using MeOH as a solvent. The filtrate was concentrated under a *vacuum* to obtain a crude product which was used for the *N*-Cbz protection reaction without purification.

To a solution of crude product in dry THF (8 mL), LiHMDS (0.4 mL, 0.44 mmol, 1.2 equiv), benzyl chloroformate (CbzCl) (0.2 mL, 0.58 mmol, 1.6 equiv) were added at 0°C. The resulting mixture was allowed to stir at the same temperature for 3 h. After completion of the reaction, the mixture was quenched with H_2_O (3 mL). The organic layer was extracted with EtOAc (2 × 15 mL), dried over anhydrous Na_2_SO_4_, and concentrated under *vacuum* to obtain an *N*-Cbz protected crude product, which was purified by flash column chromatography on silica gel to afford a pure product **14** (120 mg, 80% yield for 2 steps) TLC *R*_*f*_ (80% EtOAc/hexane = 0.2).

**[*α*]** ^**20**^_**D**_ **=** + 28.5 (*c* = 0.07, MeOH); ^**1**^ **H NMR** (400 MHz, CDCl_3_): *δ* 7.51–7.31 (m, 10H), 5.24–5.08 (m, 4H), 4.28–4.08 (m, 5H), 3.67–3.52 (m, 2H), 3.38–3.15 (m, 2H), 1.99–1.90 (m, 6H), 1.77–1.49 (m, 3H), 1.47–1.19 (m, 10H), 1.18–1.08 (m, 3H), 1.01–0.73 (m, 6H); ^**13**^ **C NMR** (101 MHz, CDCl_3_) *δ* 170.18, 170.15, 155.08, 155.07, 134.91, 128.66, 128.61, 128.44, 71.40, 69.93, 69.89, 69.45, 69.18, 48.36, 48.32, 37.30, 34.43, 33.11, 33.04, 27.81, 27.75, 27.46, 23.20, 22.64, 14.76, 14.00; **HRMS-ESI** (*m/z*) [M+H]^**+**^calcd. for C_19_H_30_NO_5_^+^ 352.2124; found 352.2114.

## Data Availability

The original contributions presented in the study are included in the article/Supplementary Material, further inquiries can be directed to the corresponding author.
