# Supplementary figures and images for: Regioselective ring opening of aziridine for synthesizing azaheterocycle
Source: Front Chem. 2023 Oct 19;11:1280633. doi: 10.3389/fchem.2023.1280633 (PMC10620703; doi:10.3389/fchem.2023.1280633)

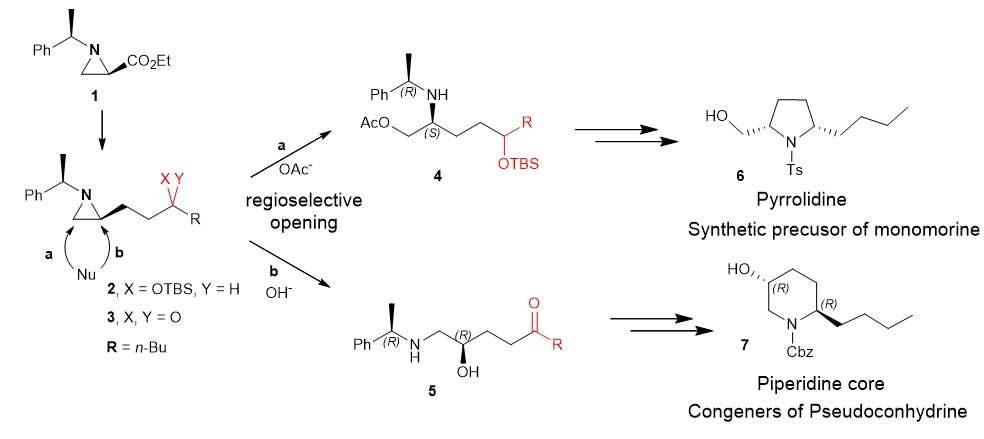

Supplement: Supplementary file 1 [file Figure2.JPEG]

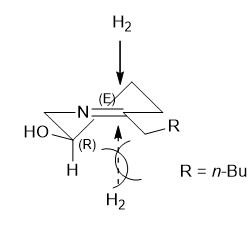

Supplement: Supplementary file 2 [file Figure5.JPEG]

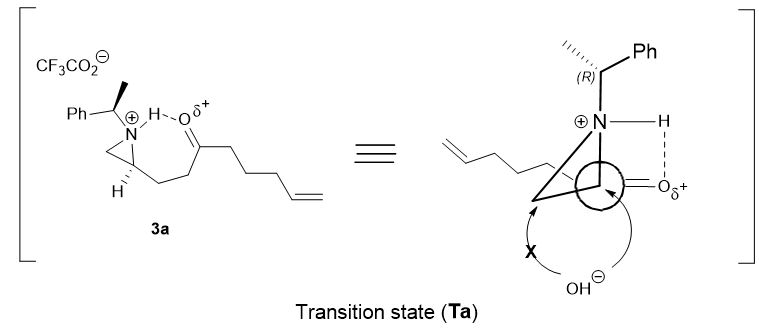

Supplement: Supplementary file 3 [file Figure3.JPEG]

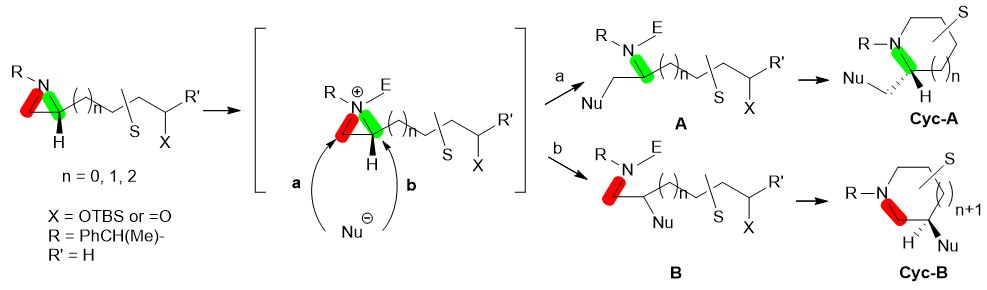

Supplement: Supplementary file 4 [file Figure1.JPEG]

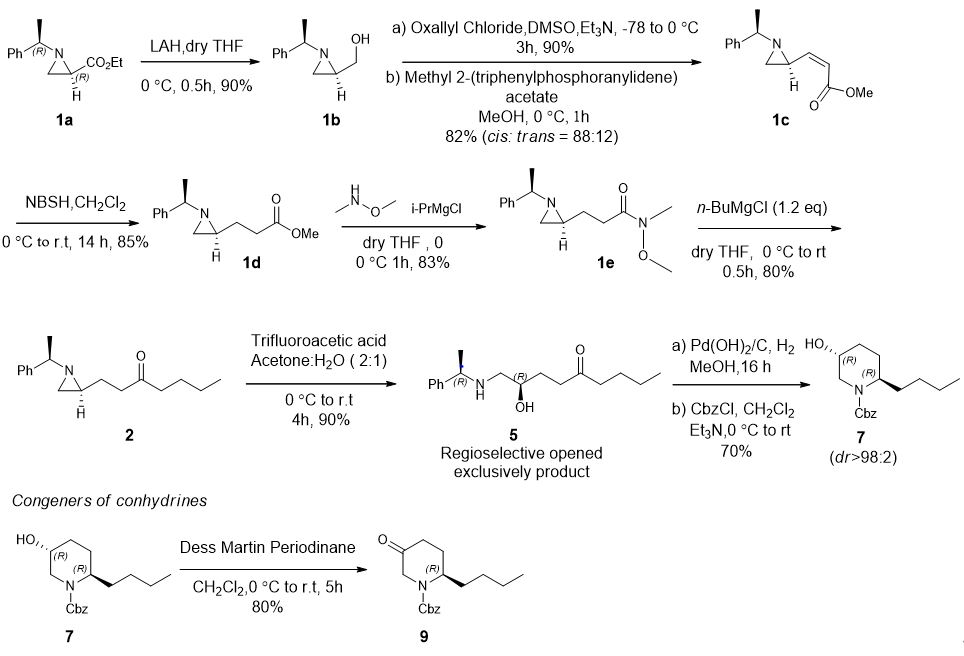

Supplement: Supplementary file 5 [file Figure4.JPEG]

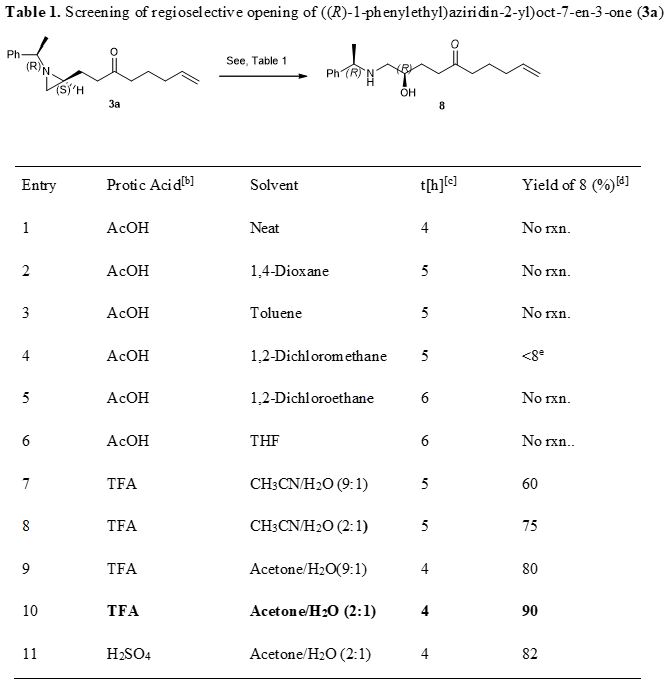

Supplement: Supplementary file 6 [file Figure8.JPEG]

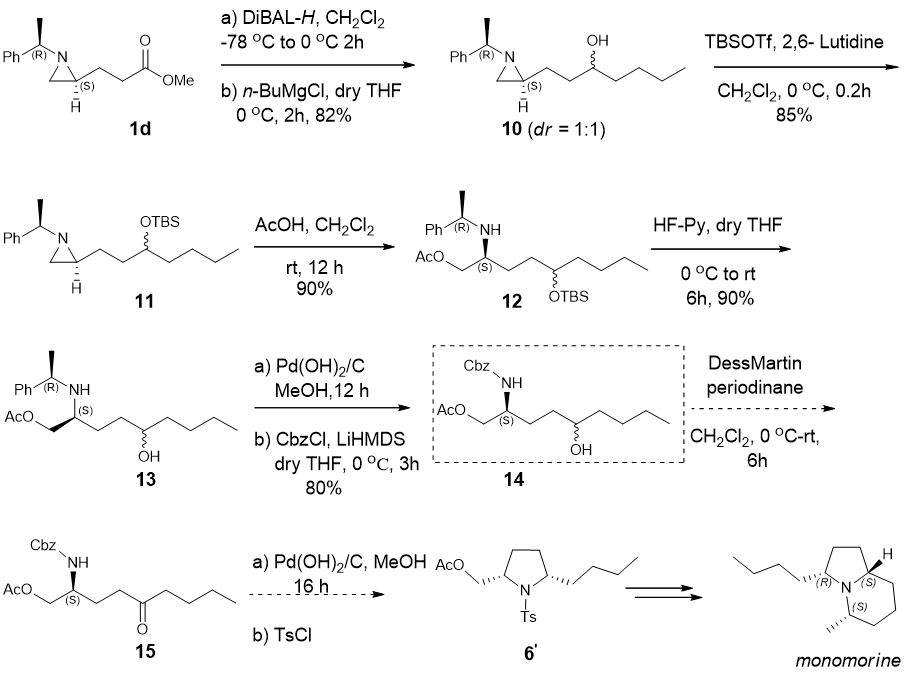

Supplement: Supplementary file 8 [file Figure7.JPEG]

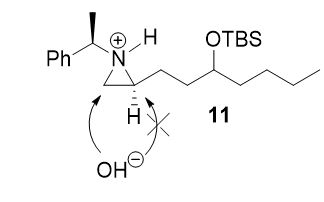

Supplement: Supplementary file 9 [file Figure6.JPEG]
