# Supplementary material for: Regioselective ring opening of aziridine for synthesizing azaheterocycle
Source: Front Chem. 2023 Oct 19;11:1280633. doi: 10.3389/fchem.2023.1280633 (PMC10620703; doi:10.3389/fchem.2023.1280633)
Supplement: Supplementary file 7 [file DataSheet1.PDF]

Supporting Information

Regioselective ring opening of aziridine for synthesizing azaheterocycle

Nikhil Srivastava, Hyun-Joon Ha*

[a]Department of Chemistry, Hankuk University of Foreign Studies, Yongin, Kyunggi-Do 17035, Korea

E-mail: hjha@hufs.ac.kr

Table of Contents

TFA-mediated ^{1}H-NMR spectral studies of **3a** S2
^{1}H and ^{13}C NMR spectra of **3a** S3
^{1}H and ^{13}C NMR spectra of **8**(under TFA) S4
^{1}H and ^{13}C NMR spectra of **8**(under Sulfuric acid) S5
^{1}H and ^{13}C NMR spectra of **1c** S6
^{1}H and ^{13}C NMR spectra of **1d** S7
^{1}H and ^{13}C NMR spectra of **1e** S8
^{1}H and ^{13}C NMR spectra of **2** S9
^{1}H and ^{13}C NMR spectra of **5** S10
^{1}H and ^{13}C NMR spectra of **7** S11
^{1}H and ^{13}C NMR spectra of **9** S12
^{1}H and ^{13}C NMR spectra of **10** S13
^{1}H and ^{13}C NMR spectra of **11** S14
^{1}H and ^{13}C NMR spectra of **12** S15
^{1}H and ^{13}C NMR spectra of **13** S16
^{1}H and ^{13}C NMR spectra of **14** S17
HRMS spectra of compound **3a-14** S18-S24

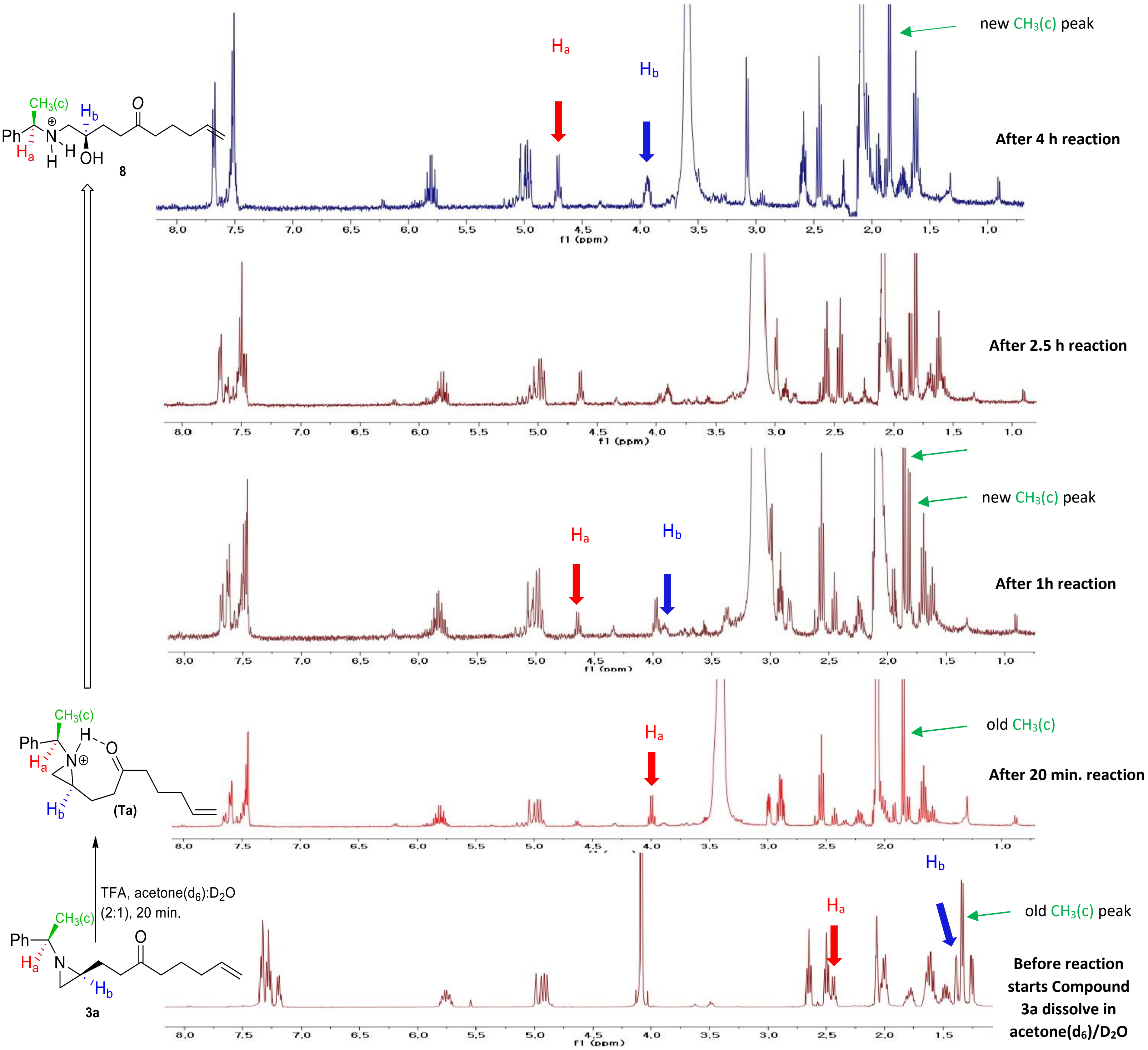

Figure 1. TFA mediated change in ^{1}H-NMR spectra during transformation of compound **3a** for the formation of intermediate transition state (**Ta**) and regioselective ring opening product (*R*)-2-hydroxy-1-(((*R*)-1-phenylmethyl)amino)dec-9-en-5-one **8** in **acetone(d$_6$)** at different time intervals (20 mins, 1h, 2.5h and 4h). Peaks with arrows are corresponding to the ^{1}H in the same colors.

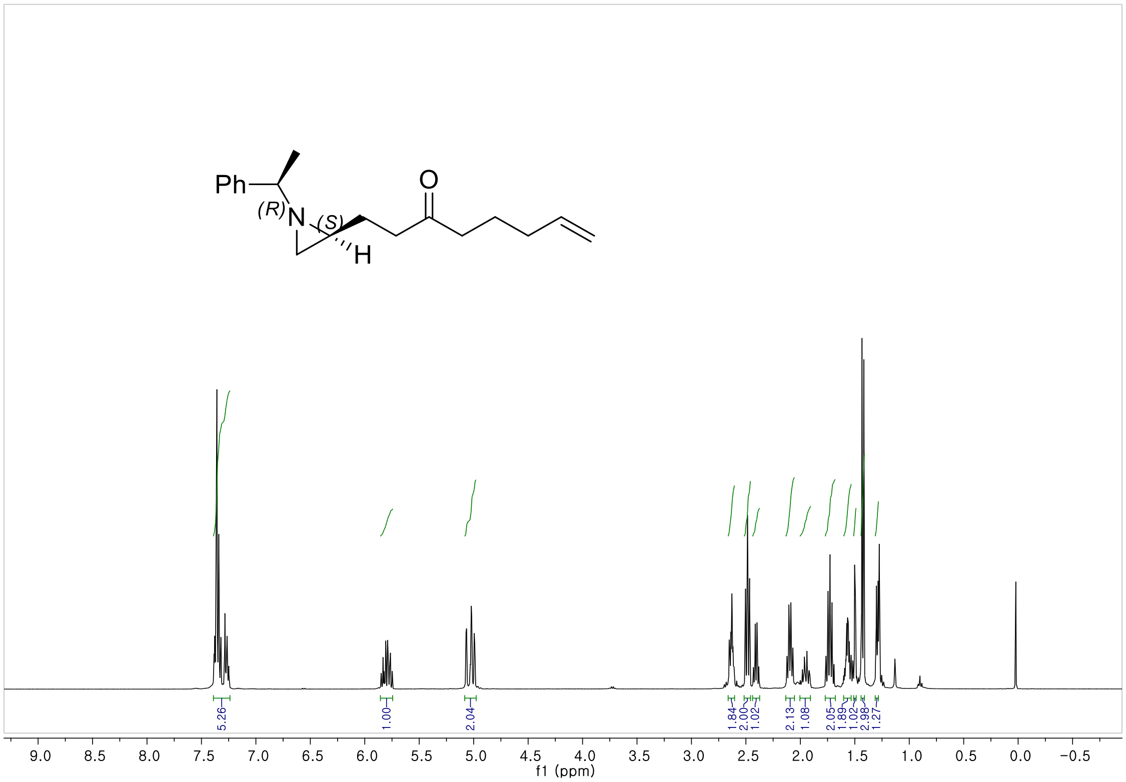

^{1}H NMR Spectra of compound **3a** (400 MHz, $CDCl_3$)

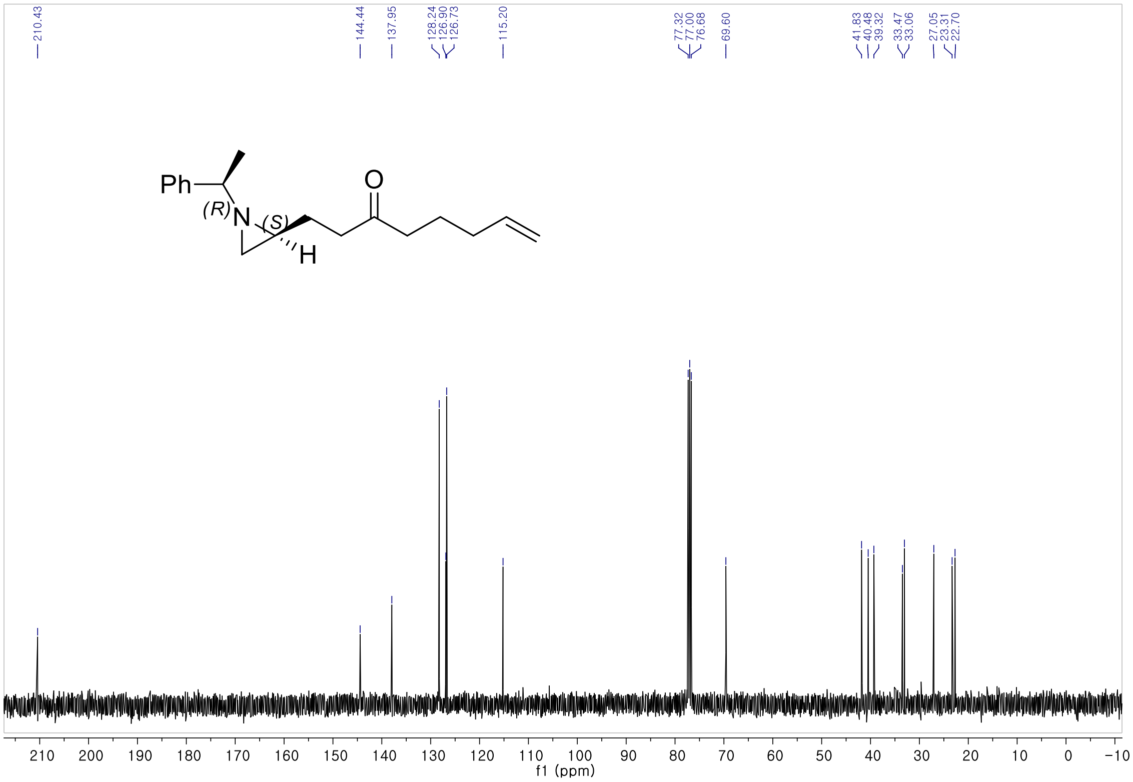

^{13}C NMR Spectra of compound **3a** (101 MHz, $CDCl_3$)

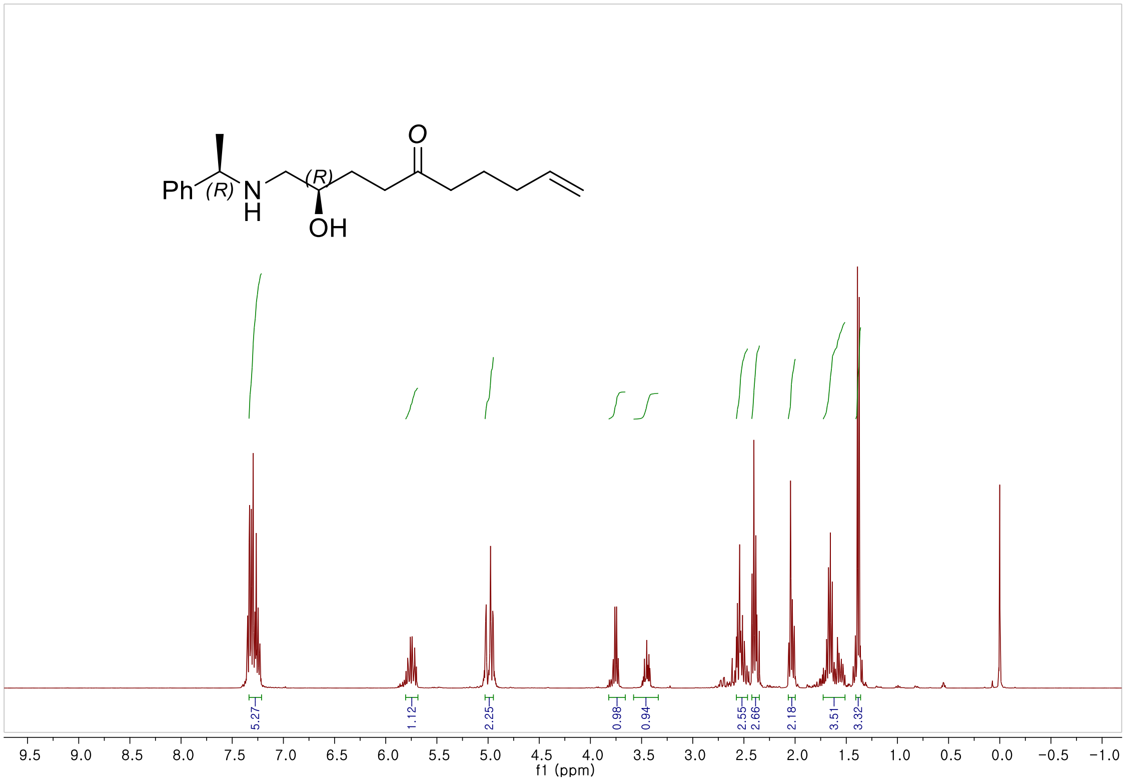

^{1}H NMR Spectra of compound **8 (**Obtained under **TFA, Table 1, entry 10)** (400 MHz, $CDCl_3$)

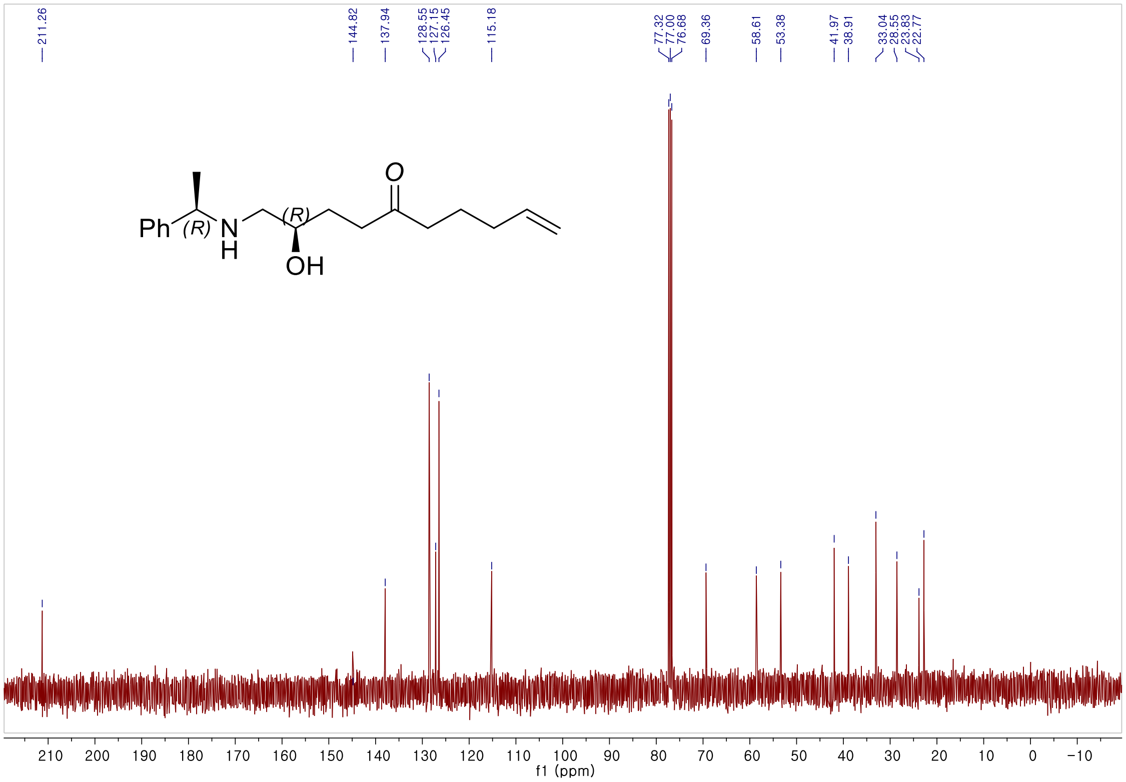

^{13}C NMR Spectra of compound **8 (**Obtained under **TFA Table 1, entry 10)** (101 MHz, $CDCl_3$)

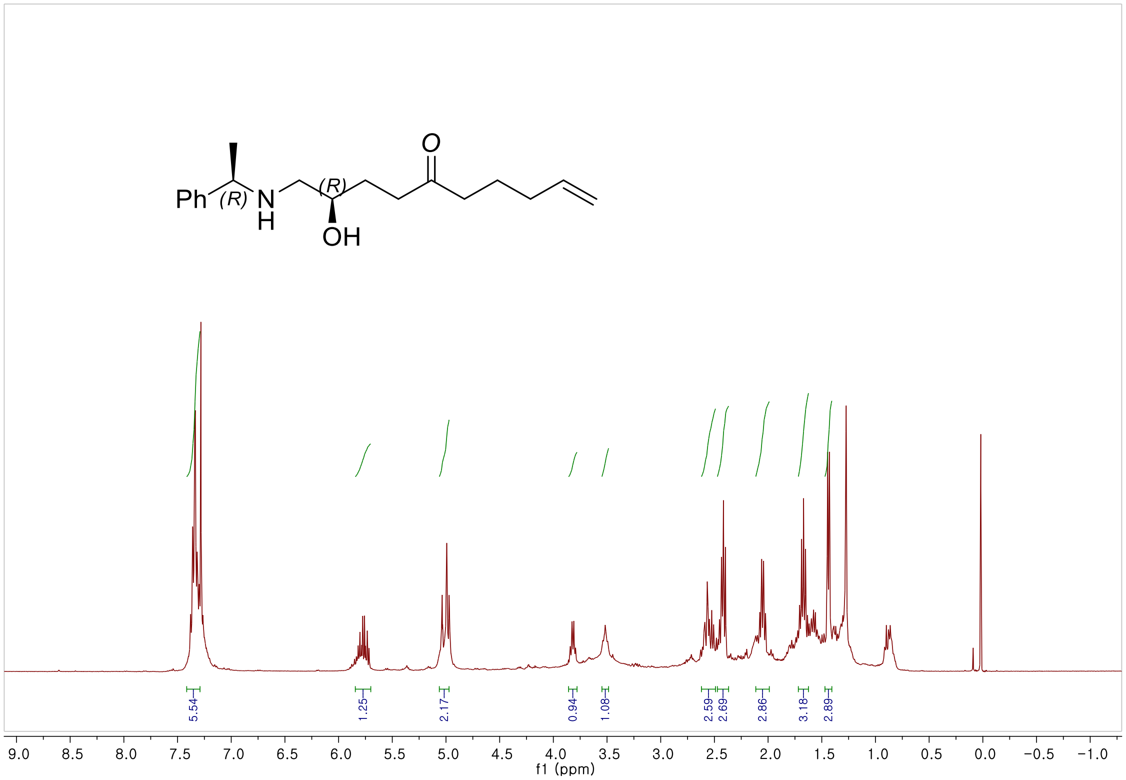

^{1}H NMR Spectra of compound **8 (**Obtained under **sulfuric acid, Table 1, entry 11)** (400 MHz, $CDCl_3$)

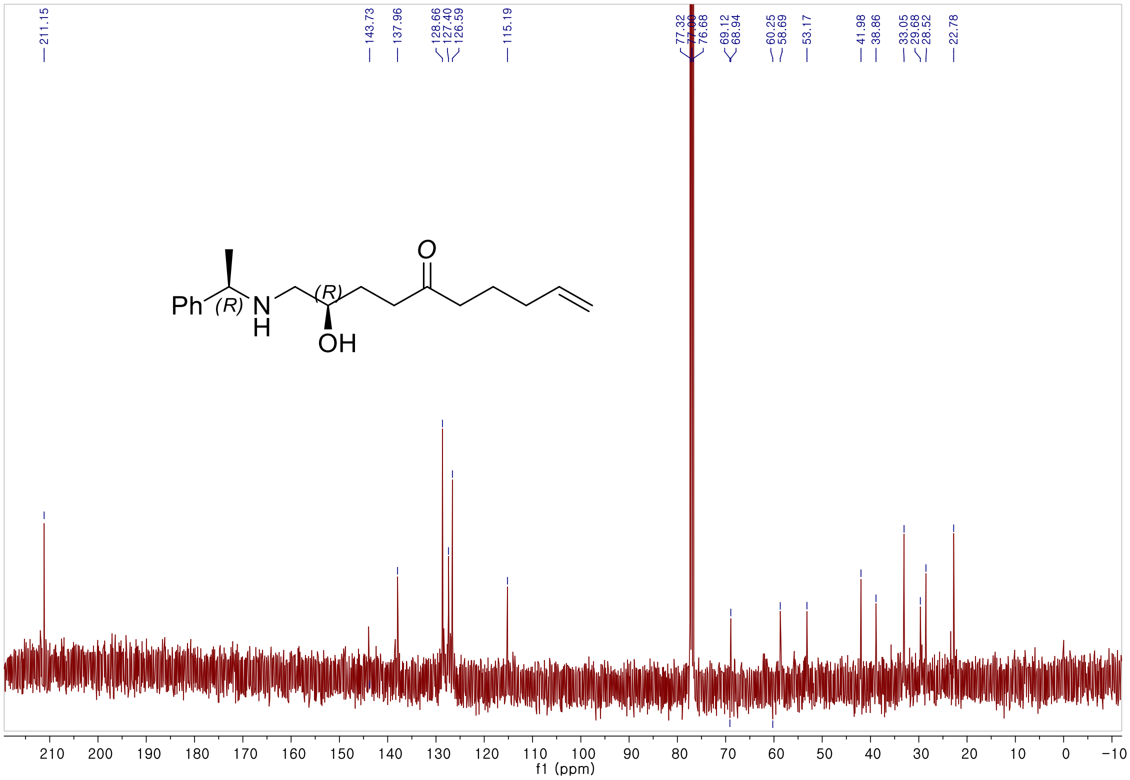

^{13}C NMR Spectra of compound **8 (**Obtained under **Sulfuric acid Table 1, entry 11)** (101 MHz, $CDCl_3$)

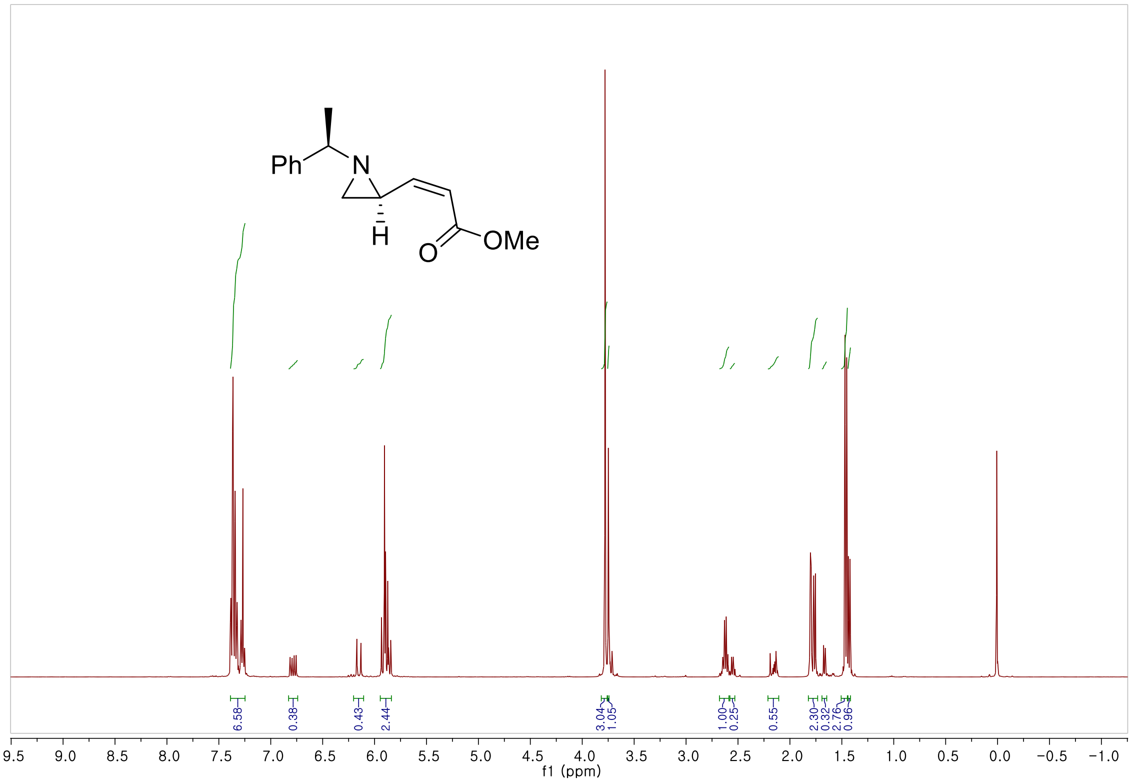

^{1}H NMR Spectra of compound **1c** (400 MHz, $CDCl_3$)

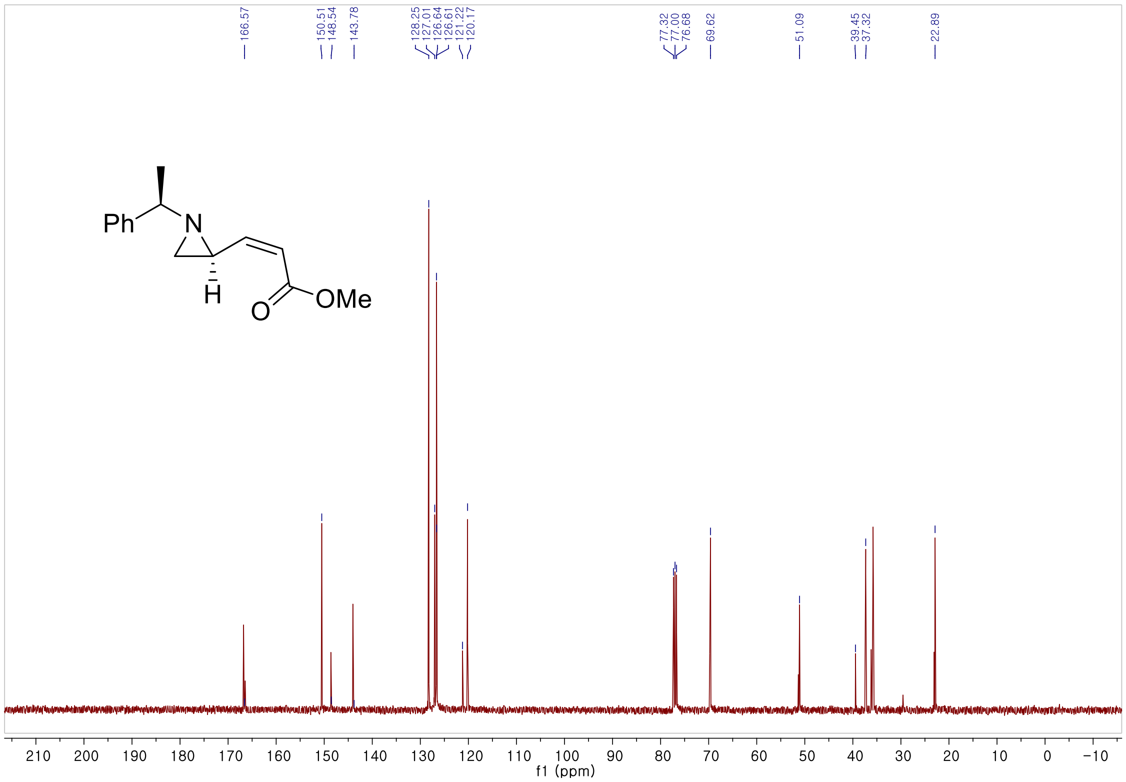

^{13}C NMR Spectra of compound **1c** (101 MHz, $CDCl_3$)

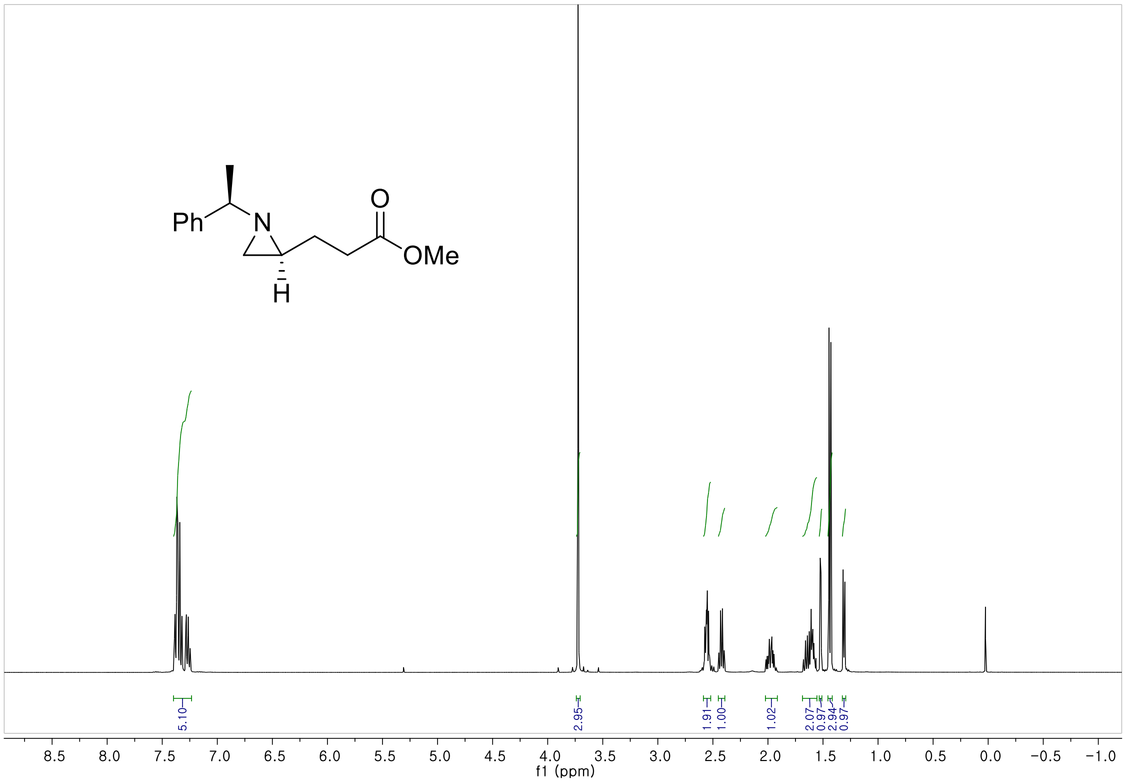

^{1}H NMR Spectra of compound **1d** (400 MHz, $CDCl_3$)

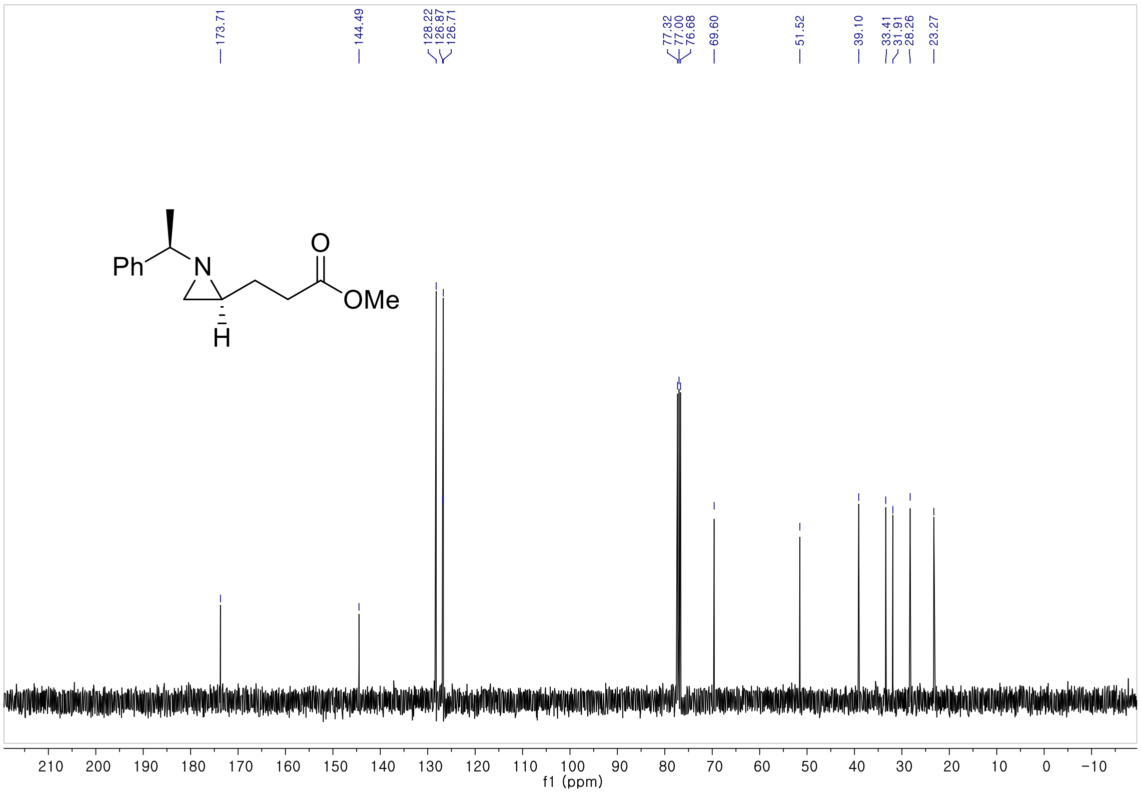

^{13}C NMR Spectra of compound **1d** (101 MHz, $CDCl_3$)

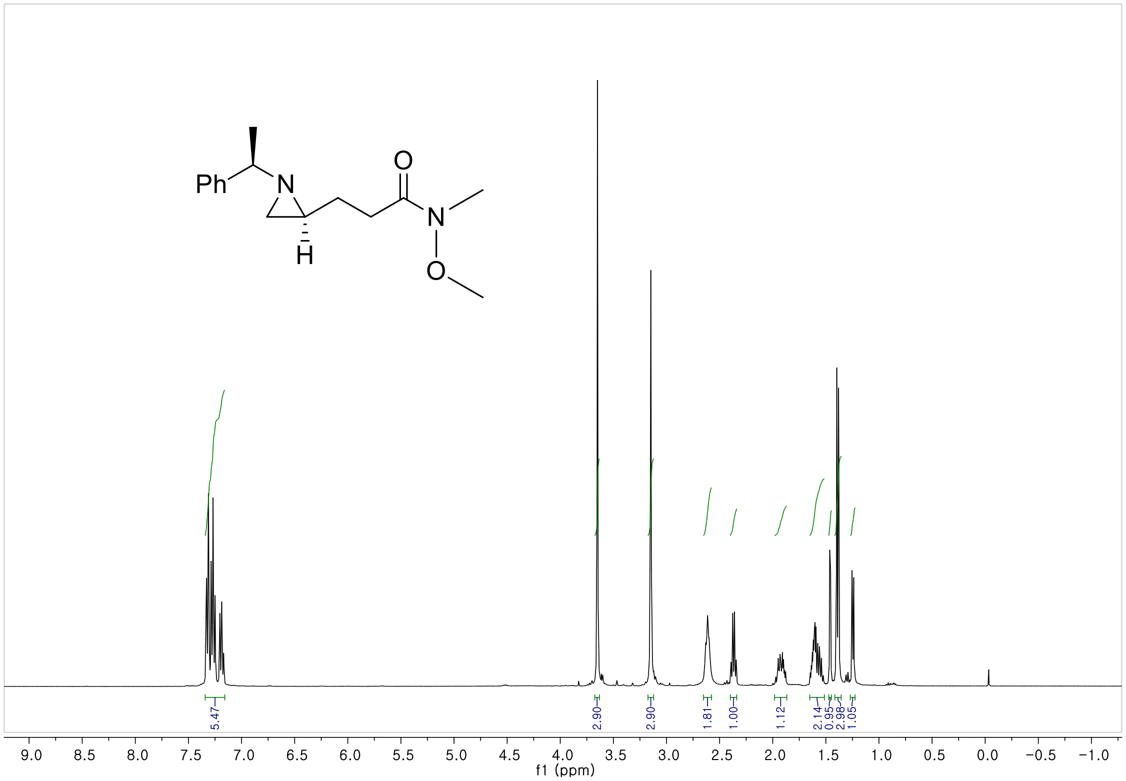

^{1}H NMR Spectra of compound **1e** (400 MHz, $CDCl_3$)

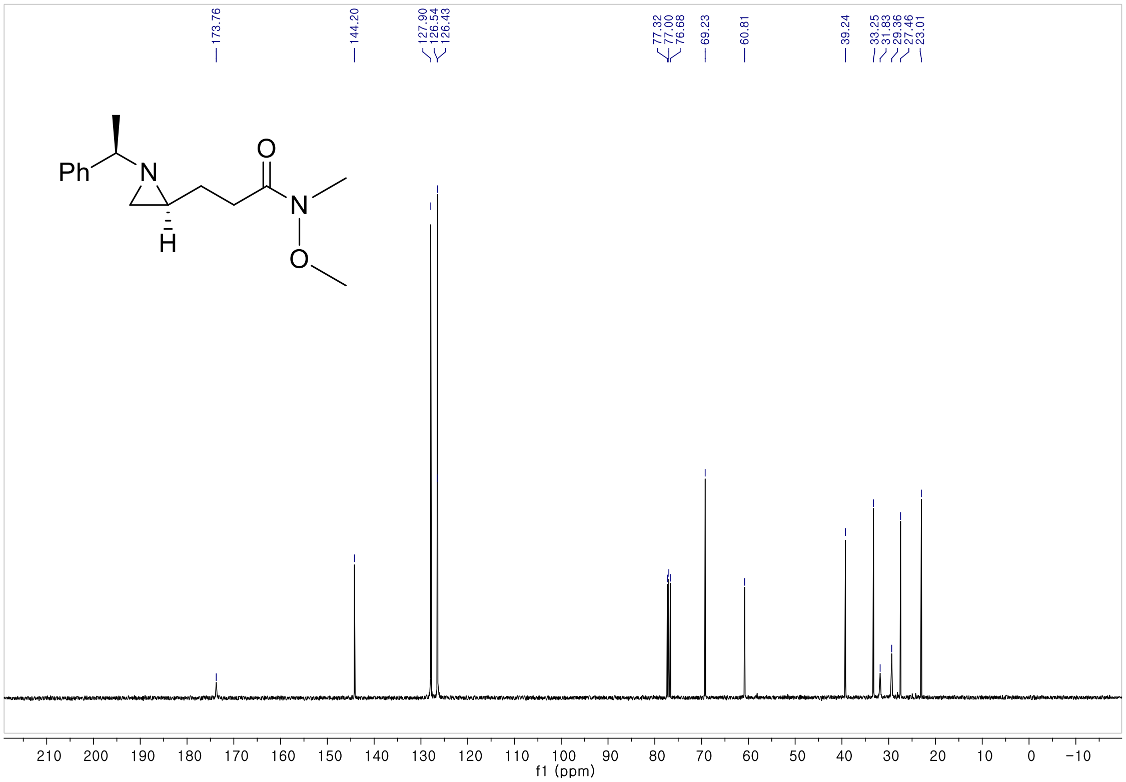

^{13}C NMR Spectra of compound **1e** (101 MHz, $CDCl_3$)

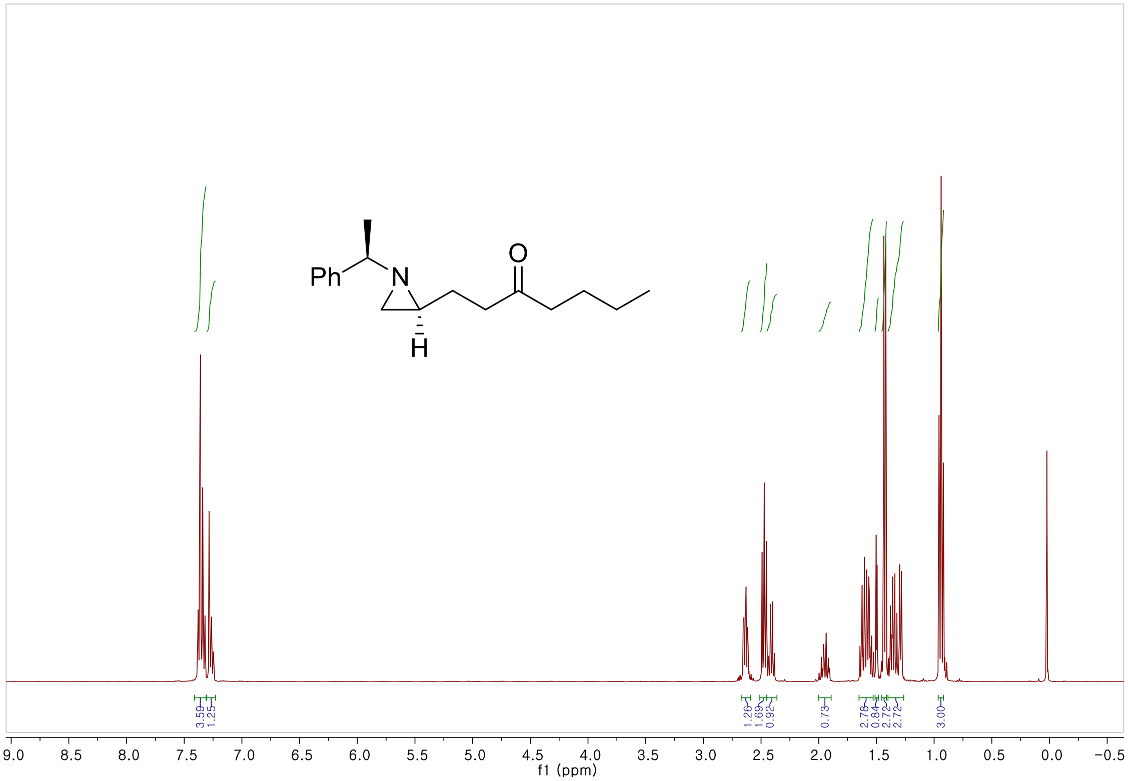

^{1}H NMR Spectra of compound **2** (400 MHz, $CDCl_3$)

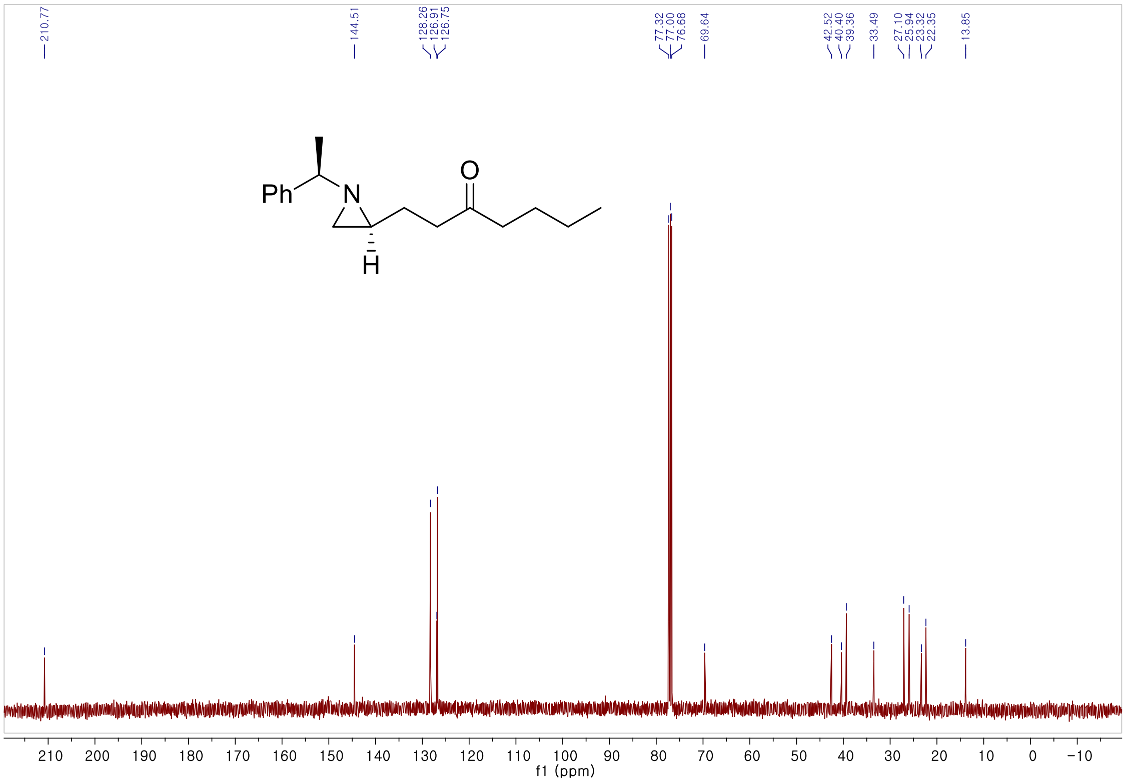

^{13}C NMR Spectra of compound **2** (101 MHz, $CDCl_3$)

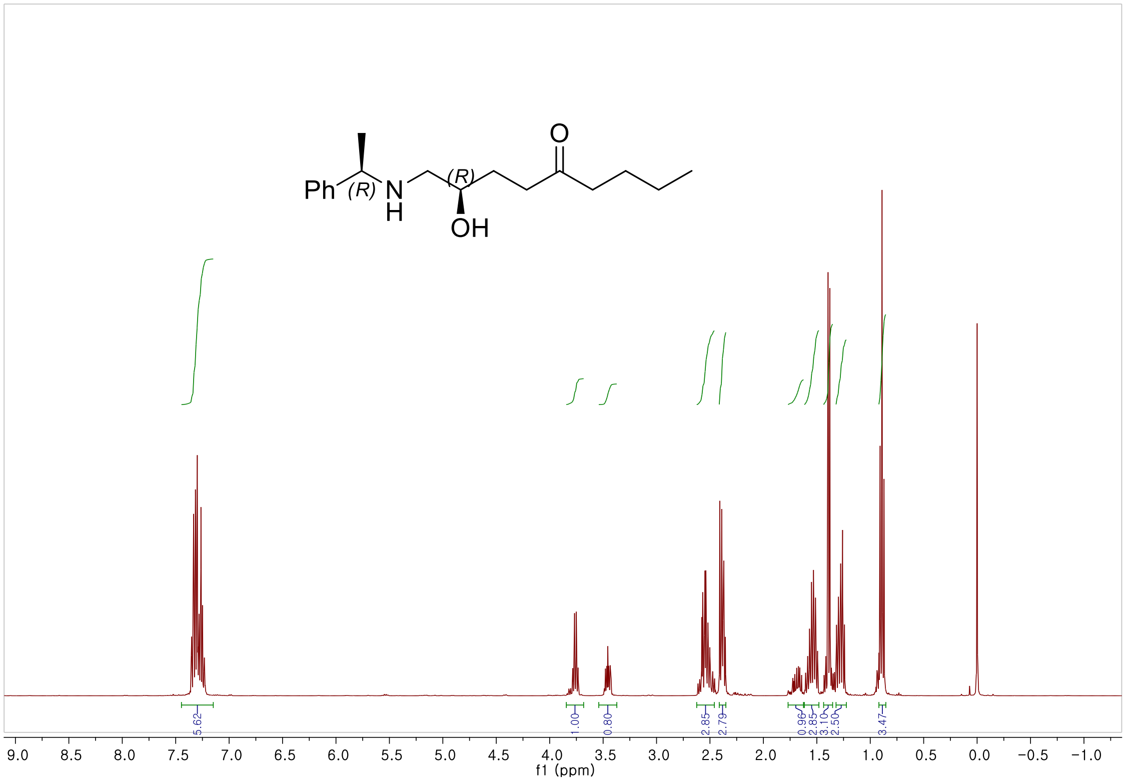

^{1}H NMR Spectra of compound **5** (400 MHz, $CDCl_3$)

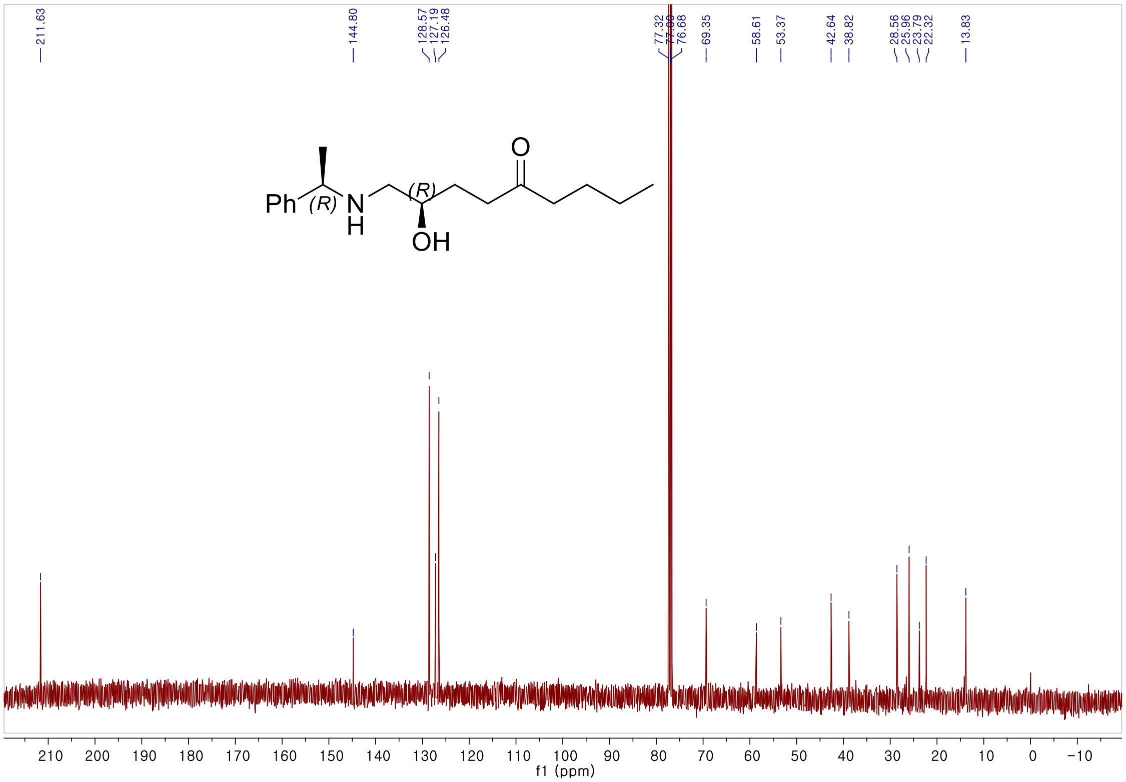

^{13}C NMR Spectra of compound **5** (101 MHz, $CDCl_3$)

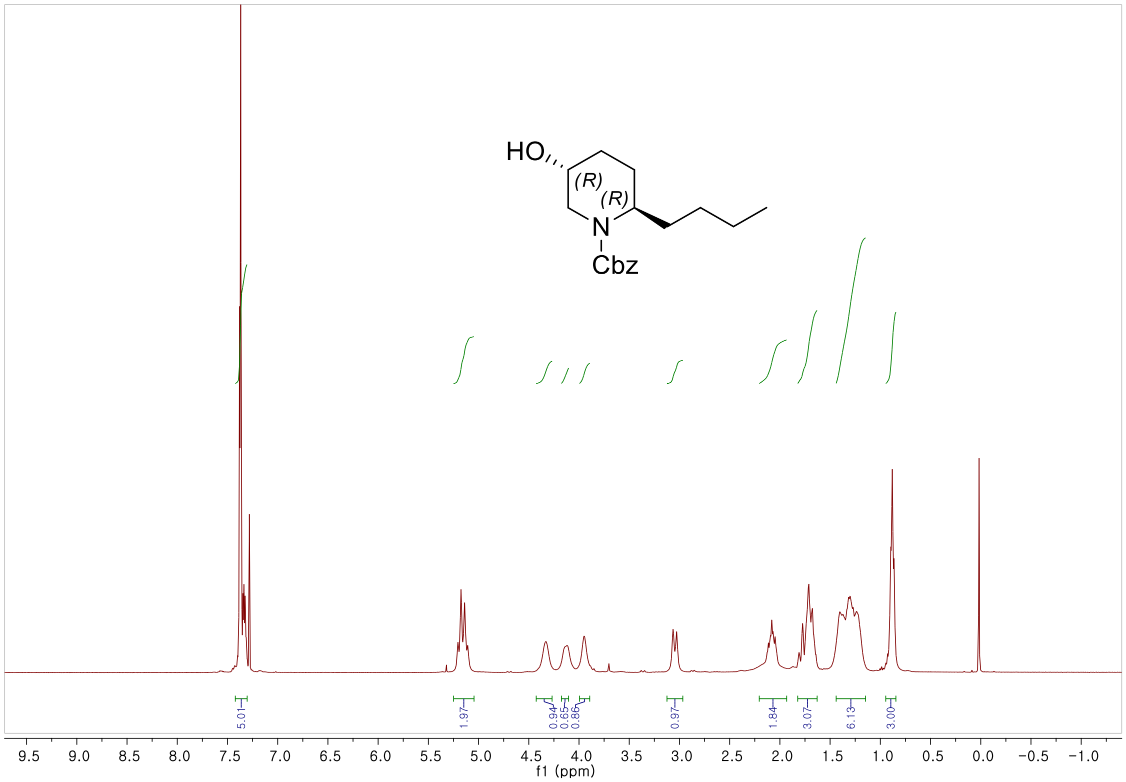

^{1}H NMR Spectra of compound **7** (400 MHz, $CDCl_3$)

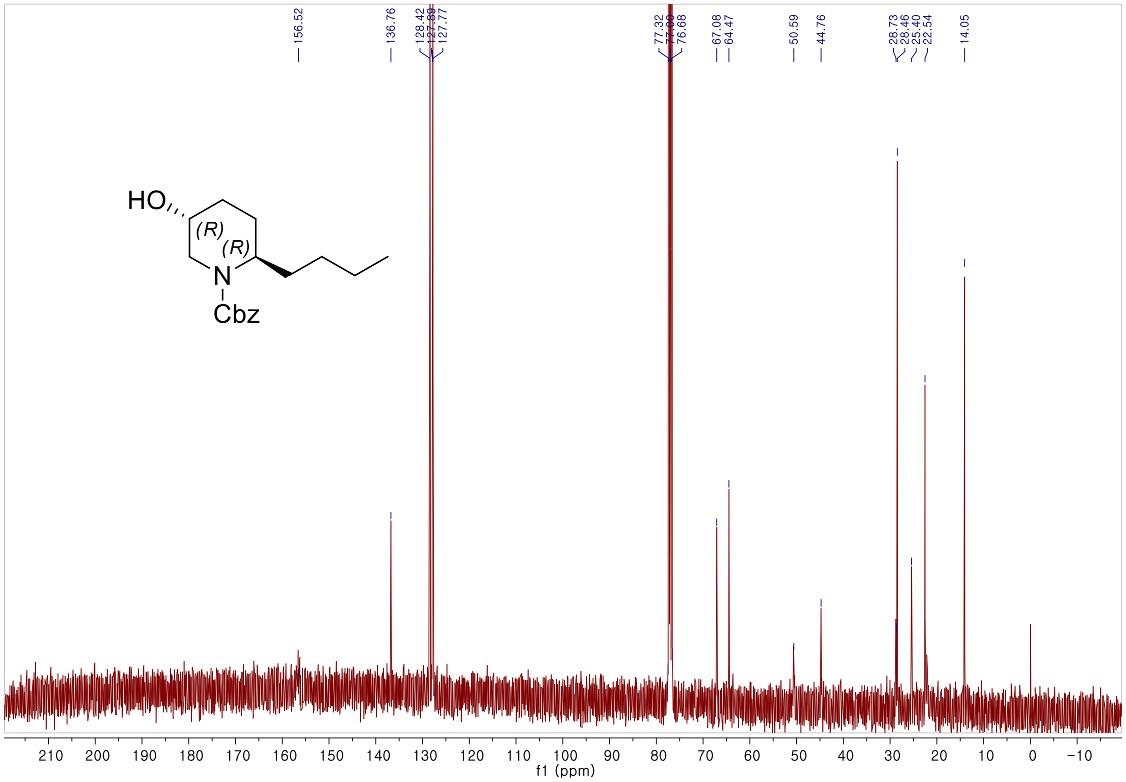

^{13}C NMR Spectra of compound **7** (101 MHz, $CDCl_3$)

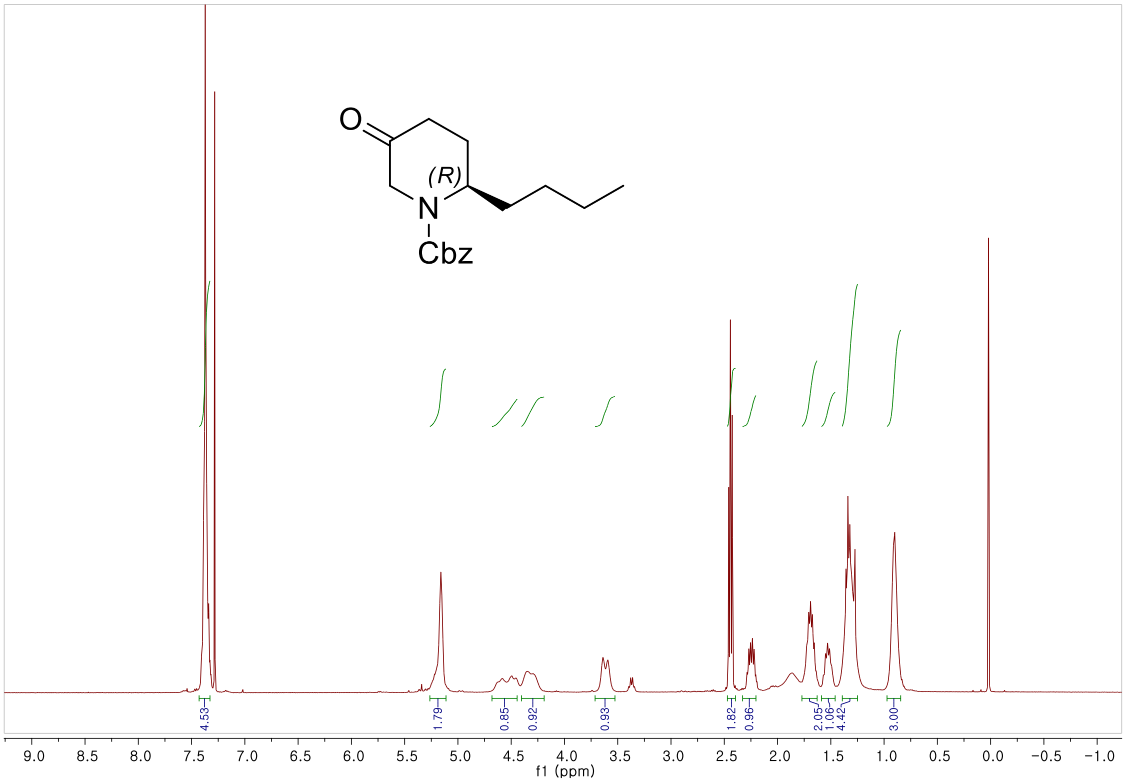

^{1}H NMR Spectra of compound **9** (400 MHz, $CDCl_3$)

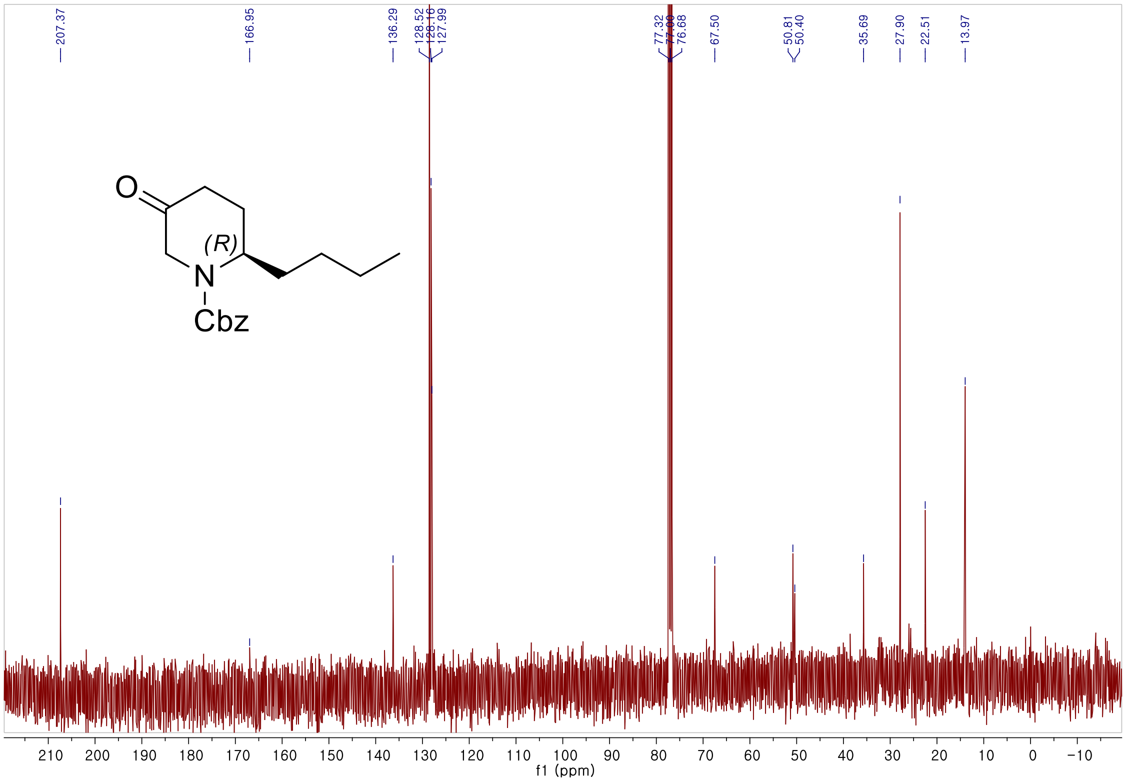

^{13}C NMR Spectra of compound **9** (101 MHz, $CDCl_3$)

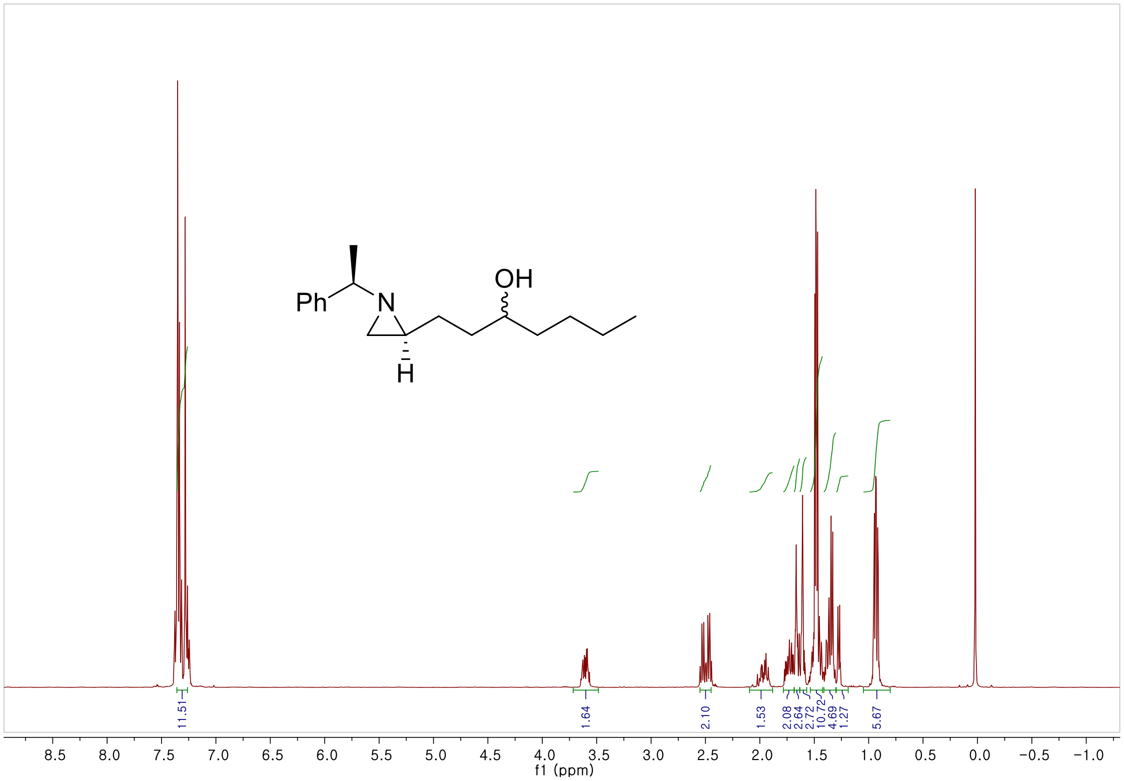

^{1}H NMR Spectra of compound **10** (400 MHz, $CDCl_3$)

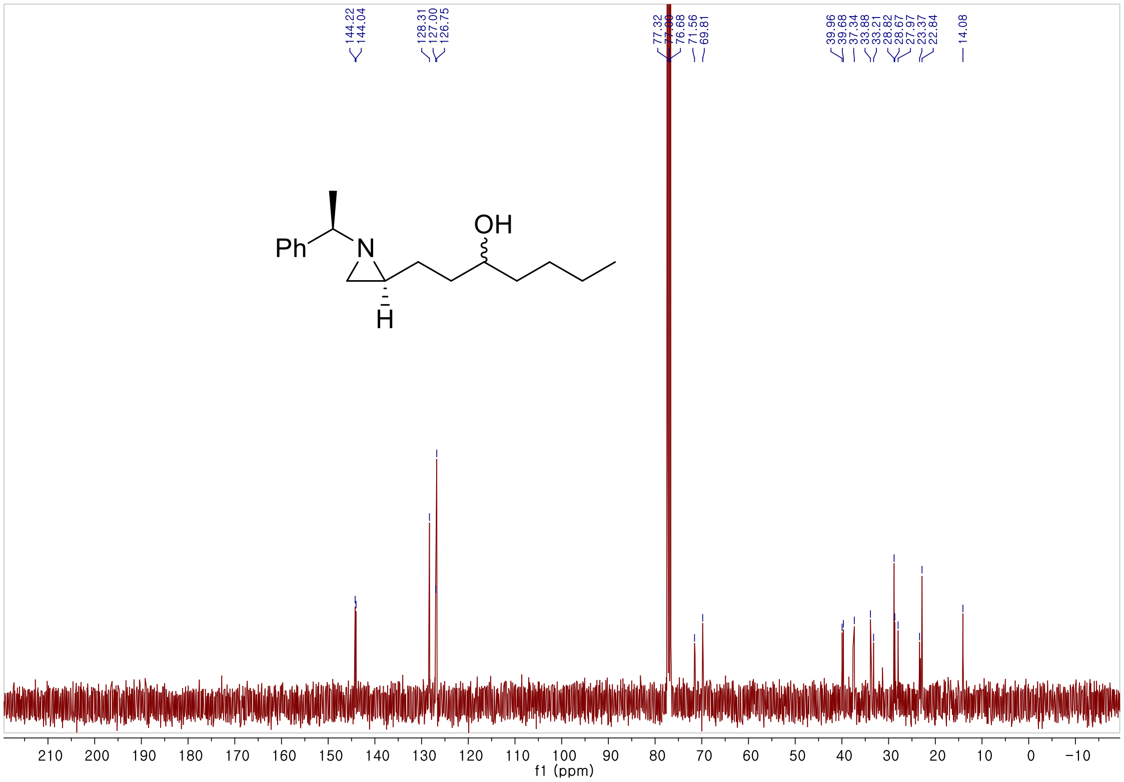

^{13}C NMR Spectra of compound **10** (101 MHz, $CDCl_3$)

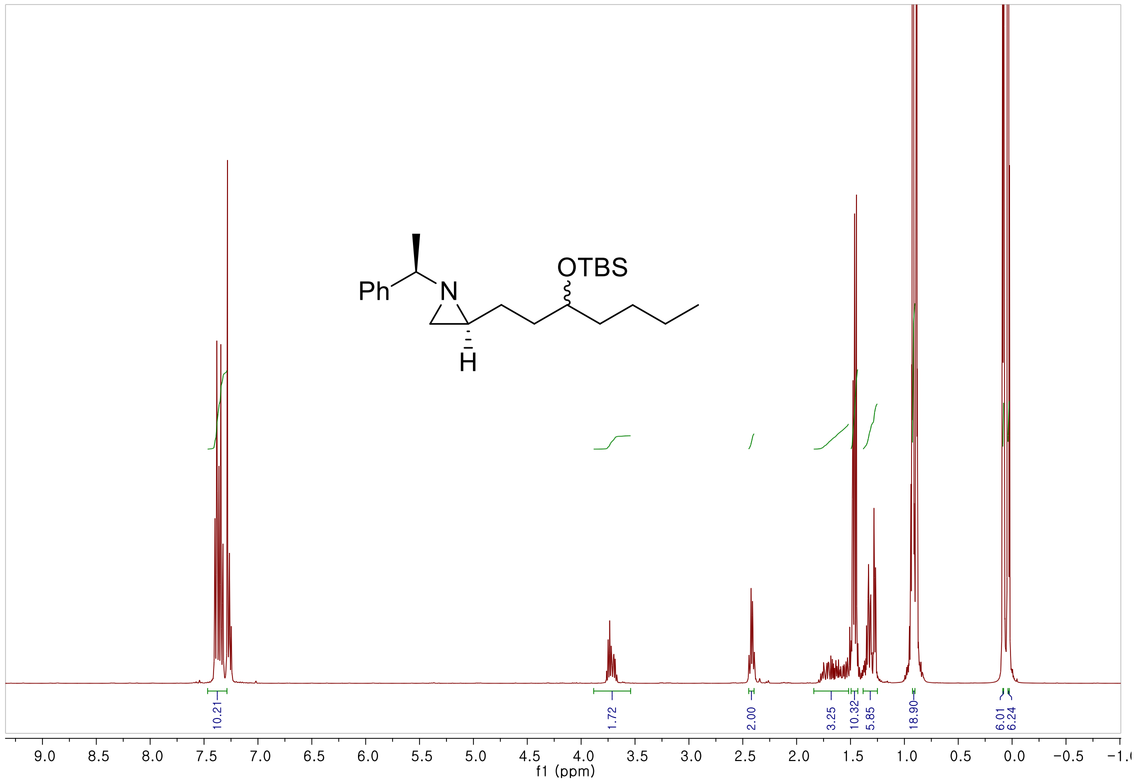

^{1}H NMR Spectra of compound **11** (400 MHz, $CDCl_3$)

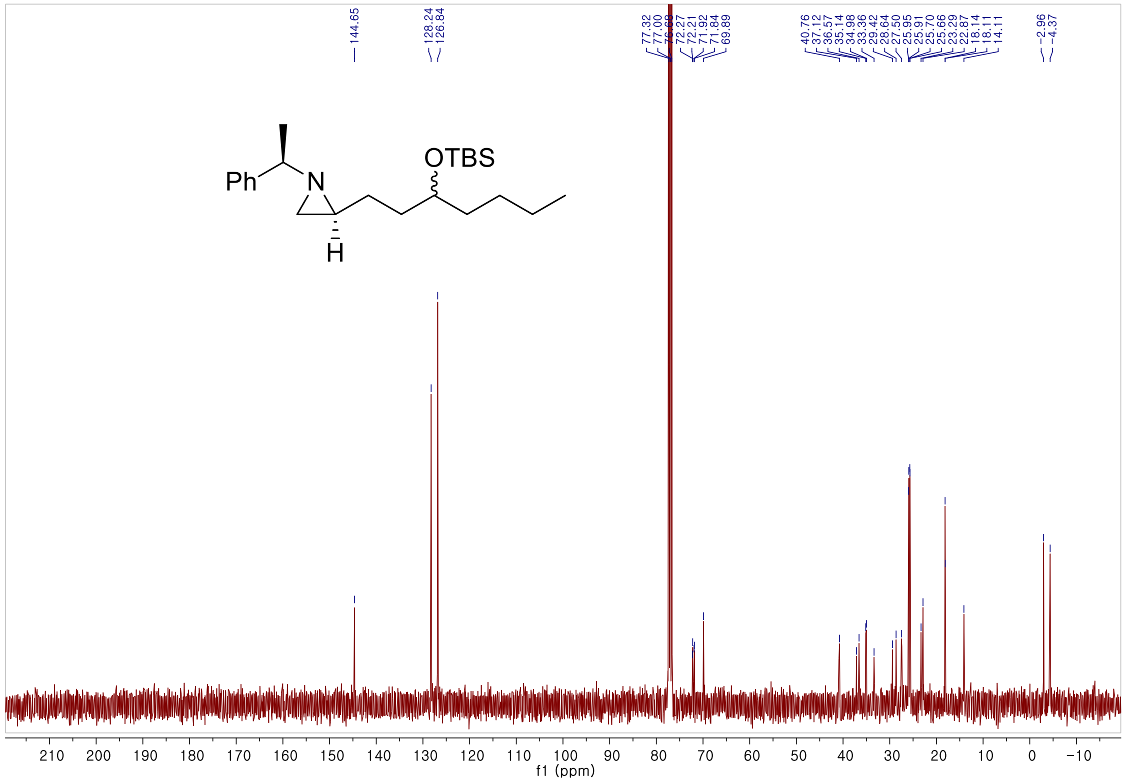

^{13}C NMR Spectra of compound **11** (101 MHz, $CDCl_3$)

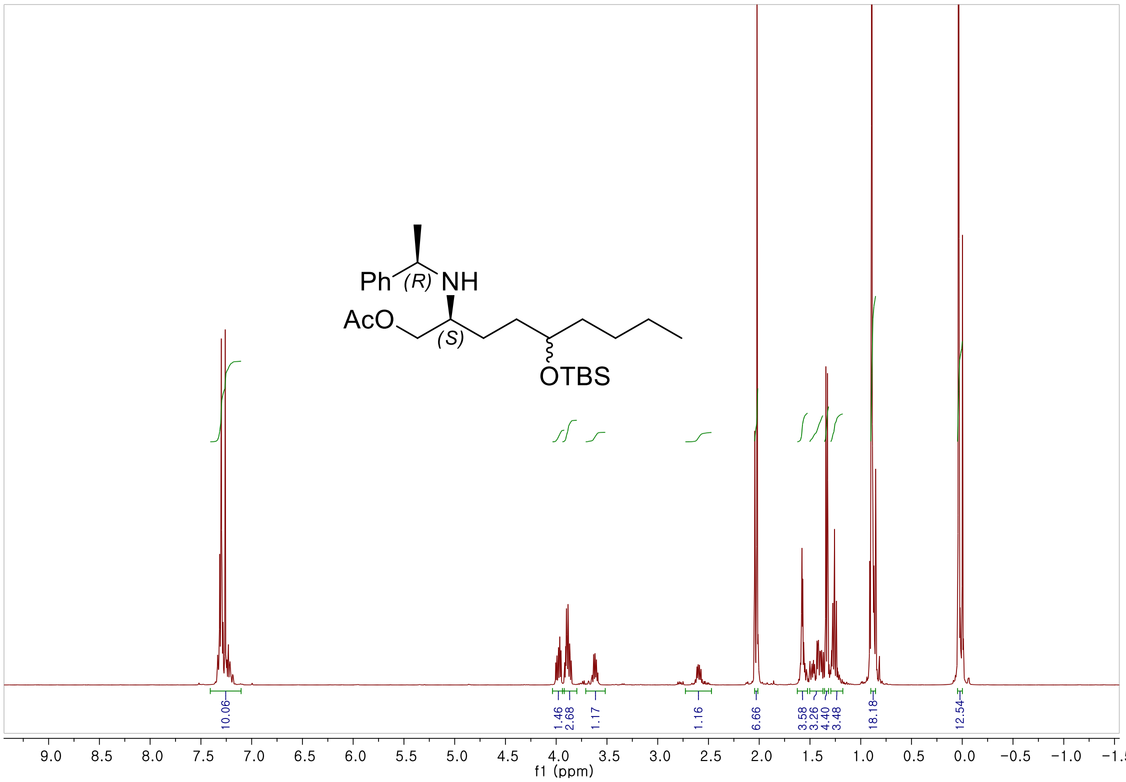

^{1}H NMR Spectra of compound **12** (400 MHz, $CDCl_3$)

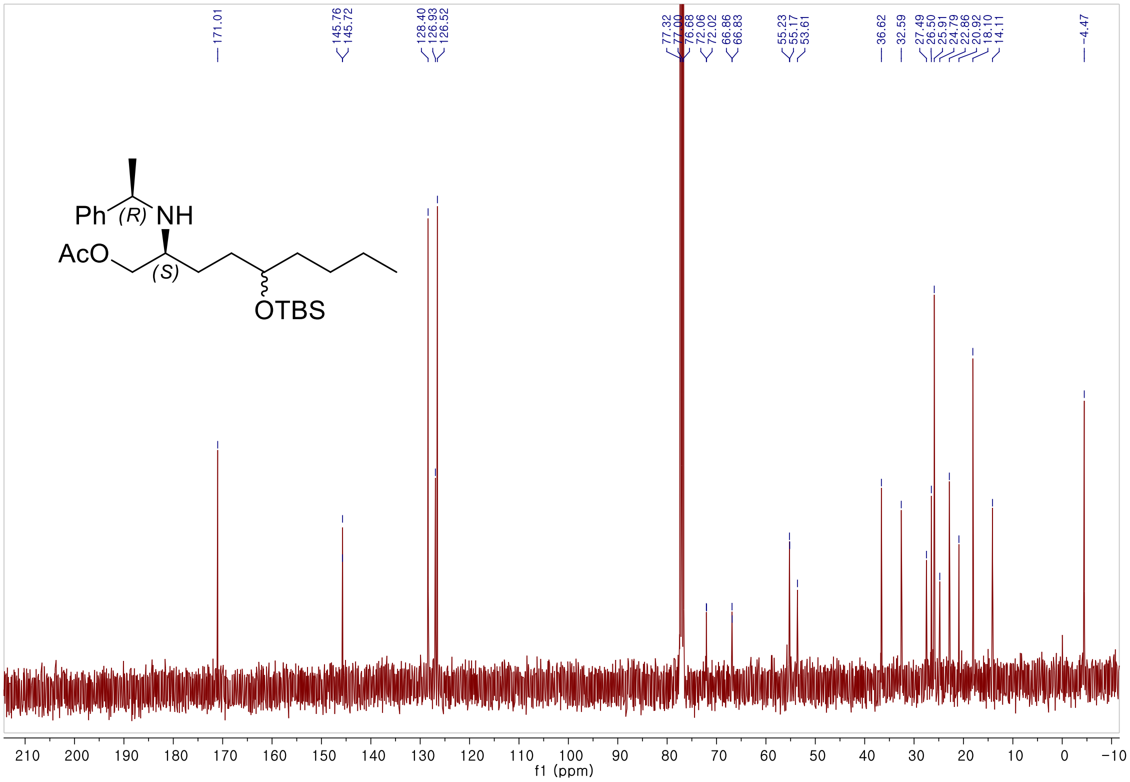

^{13}C NMR Spectra of compound **12** (101 MHz, $CDCl_3$)

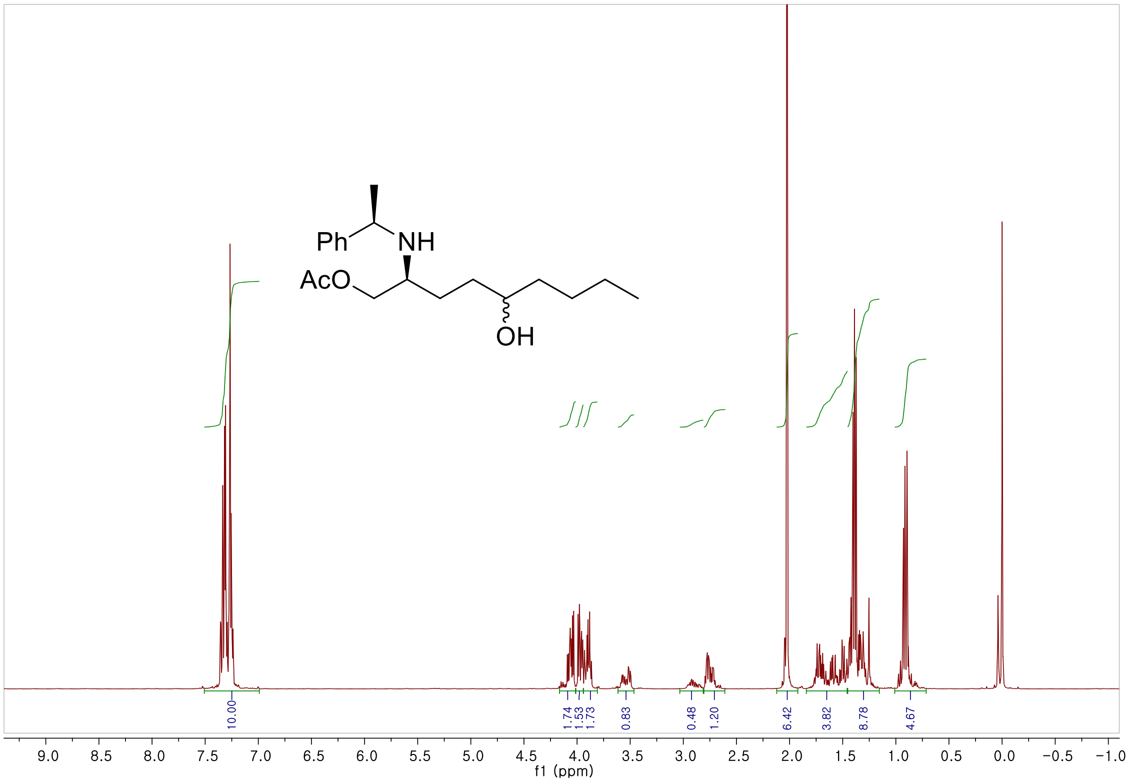

^{1}H NMR Spectra of compound **13** (400 MHz, $CDCl_3$)

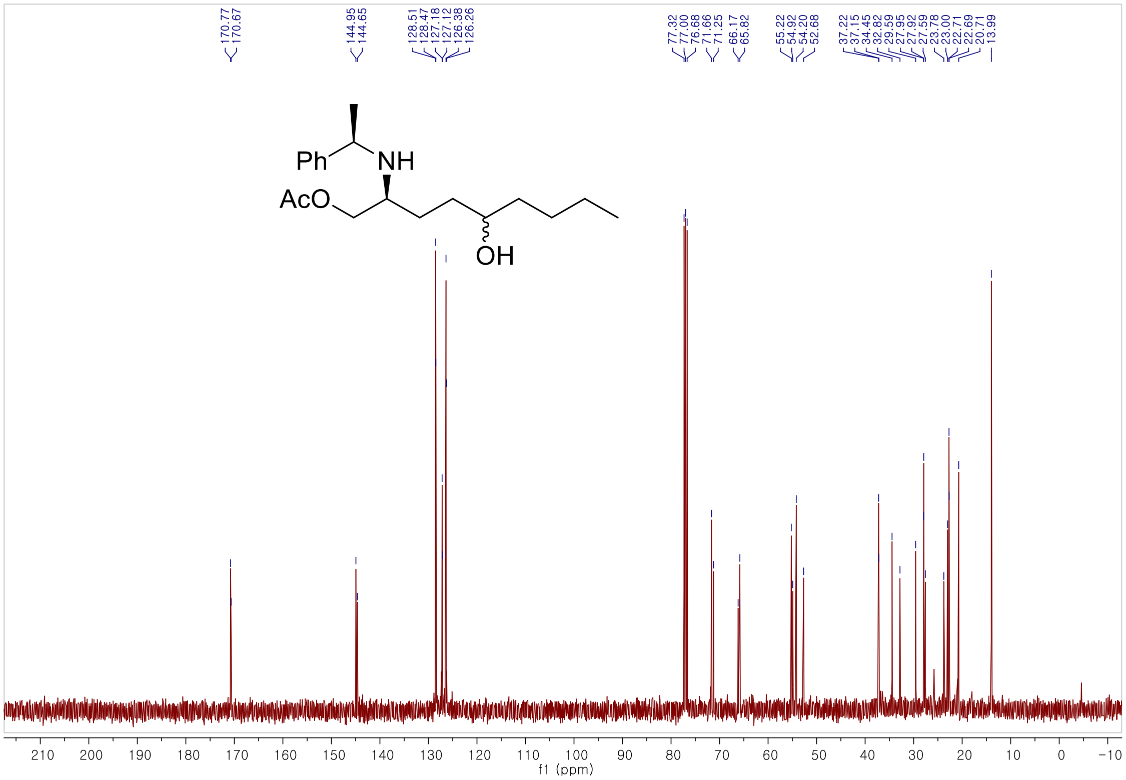

^{13}C NMR Spectra of compound **13** (101 MHz, $CDCl_3$)

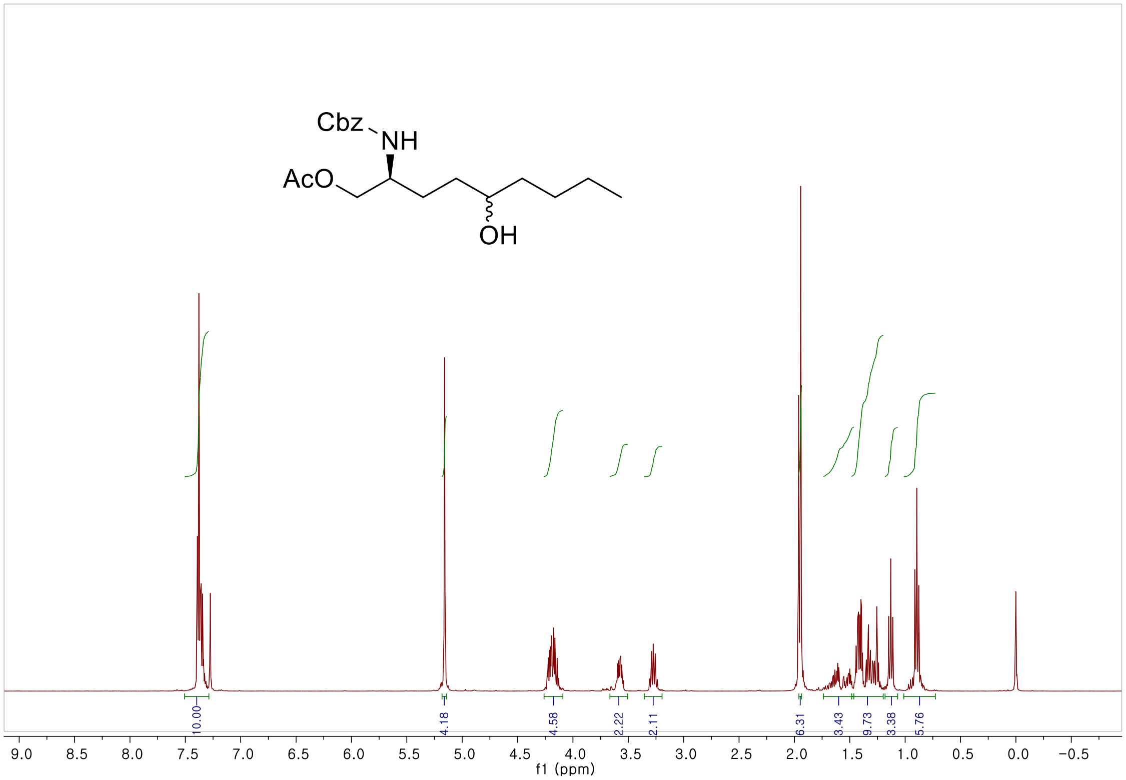

^{1}H NMR Spectra of compound **14** (400 MHz, $CDCl_3$)

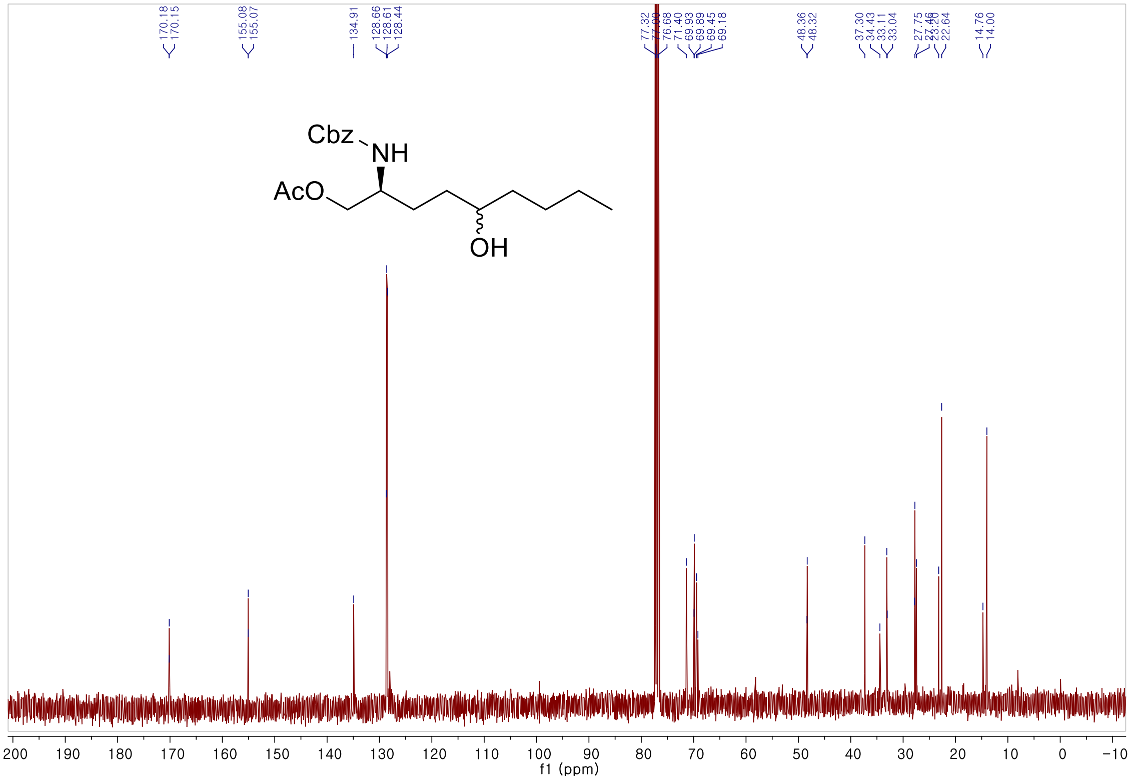

^{13}C NMR Spectra of compound **14** (101 MHz, $CDCl_3$)

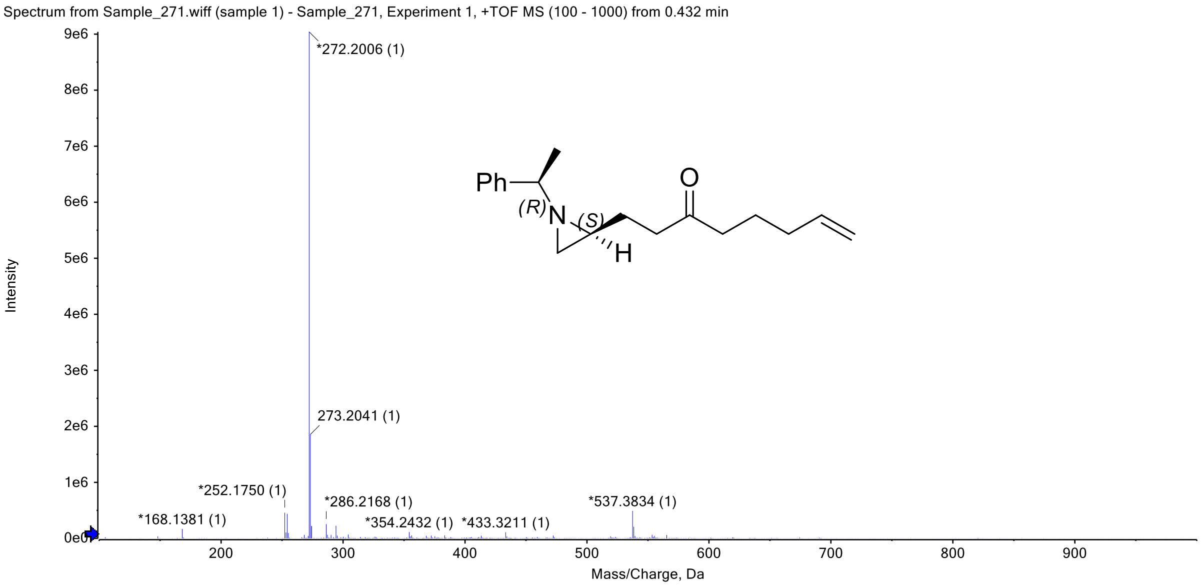

HRMS of compound **3a**

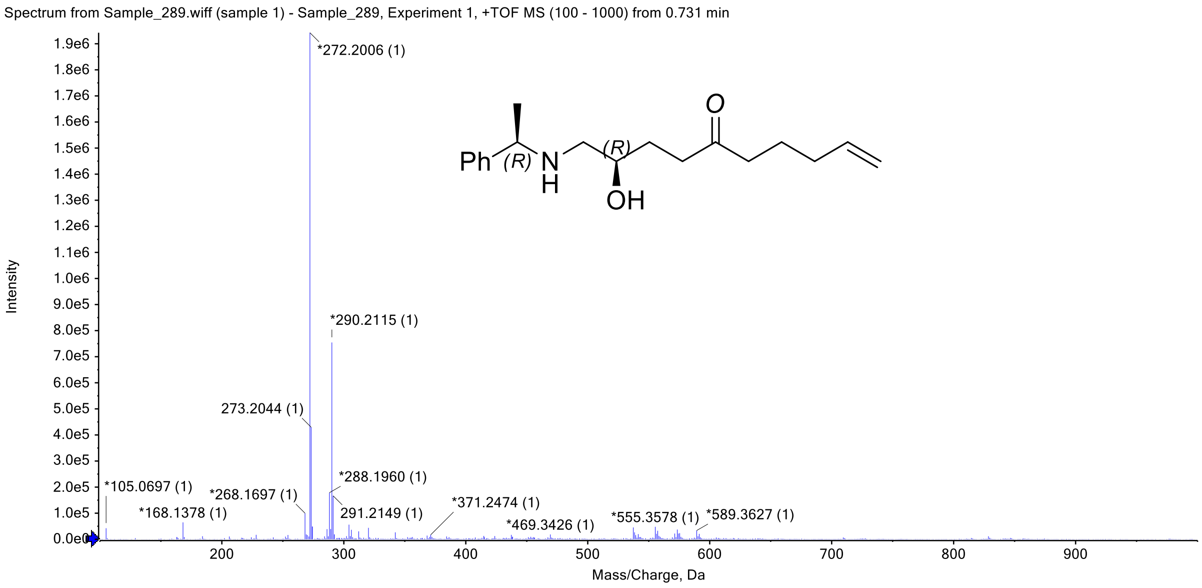

HRMS of compound **8**

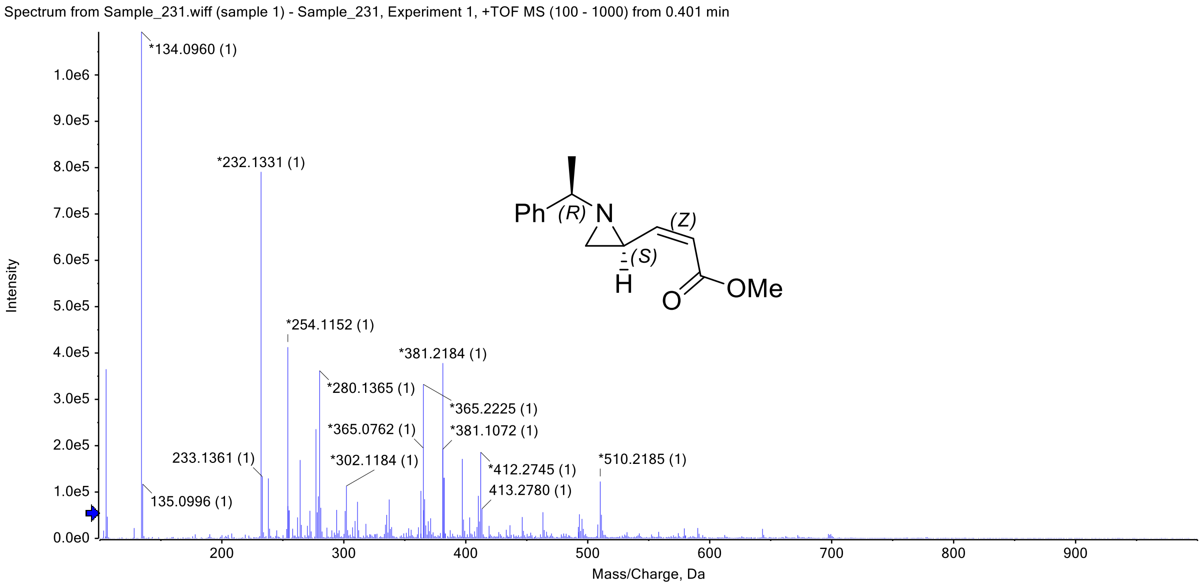

HRMS of compound **1c**

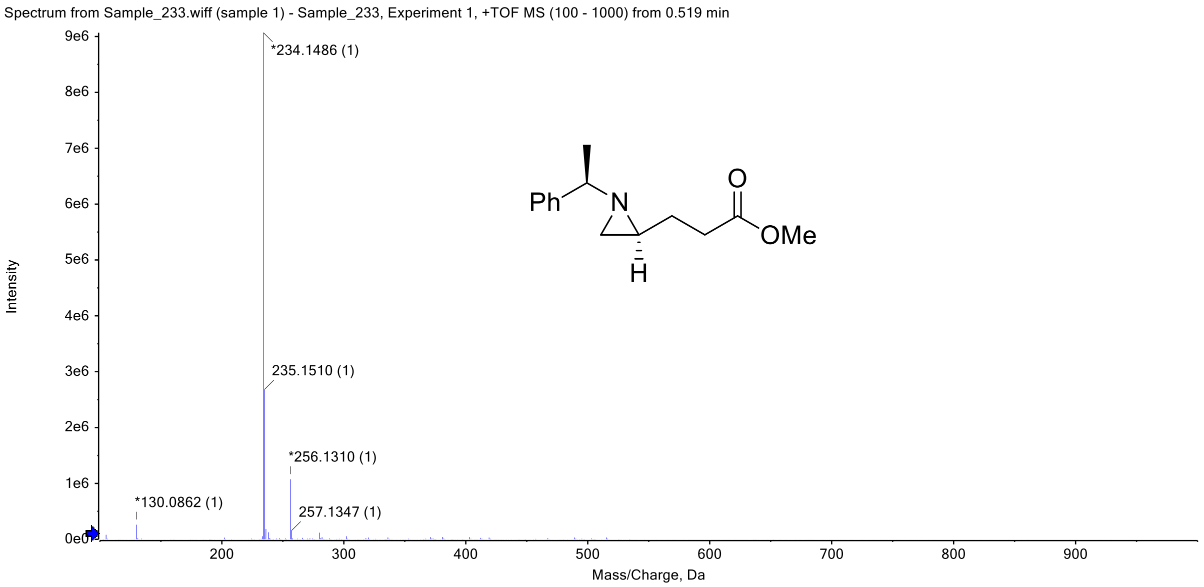

HRMS of compound **1d**

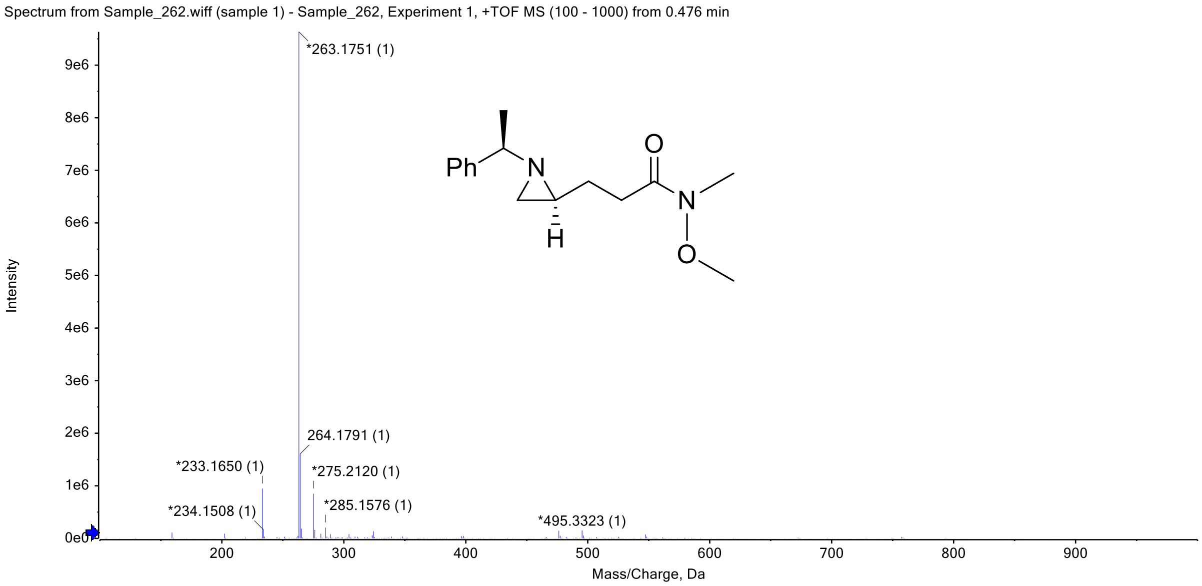

HRMS of compound **1e**

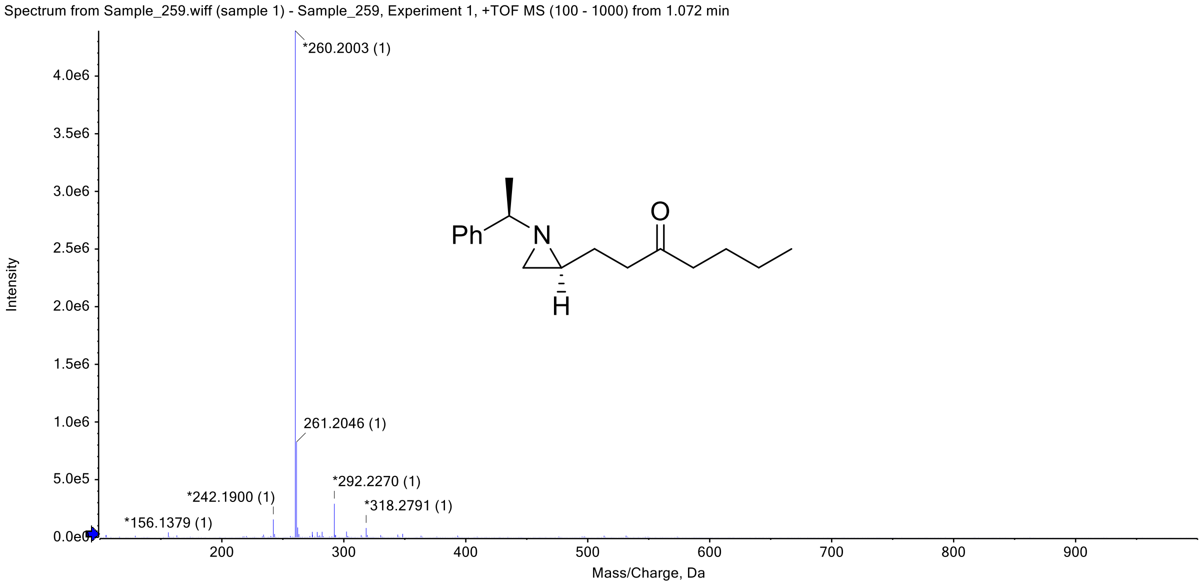

HRMS of compound **2**

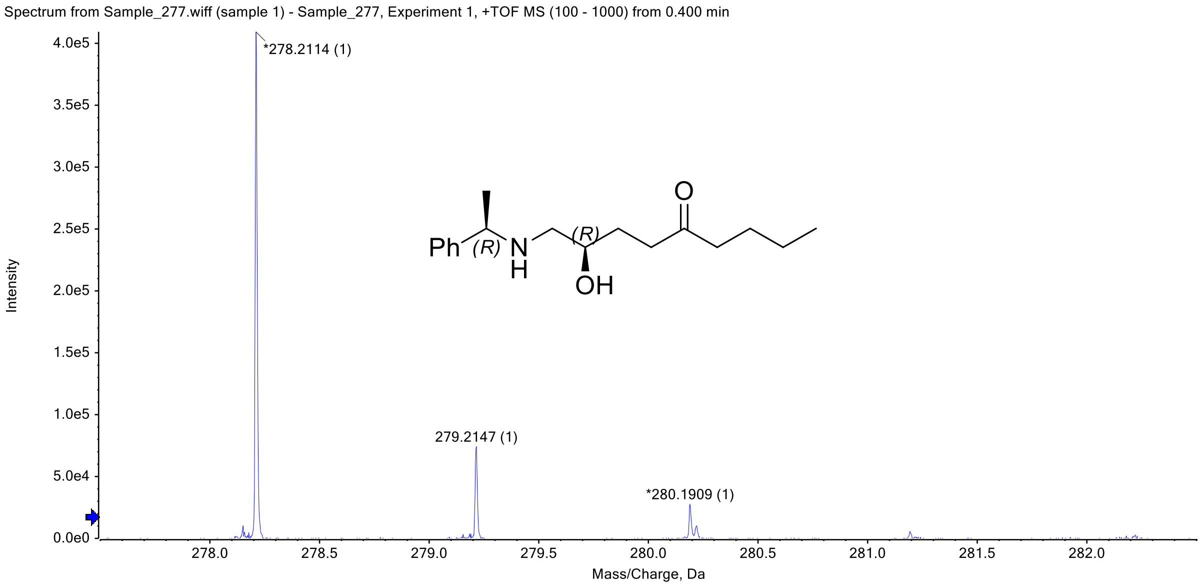

HRMS of compound **5**

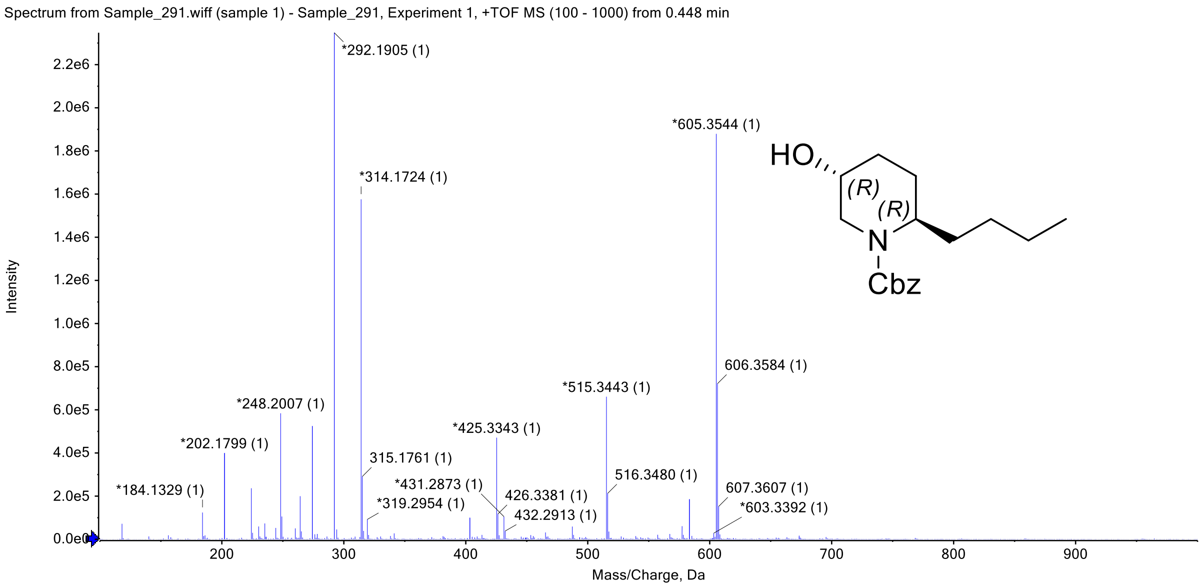

HRMS of compound **7**

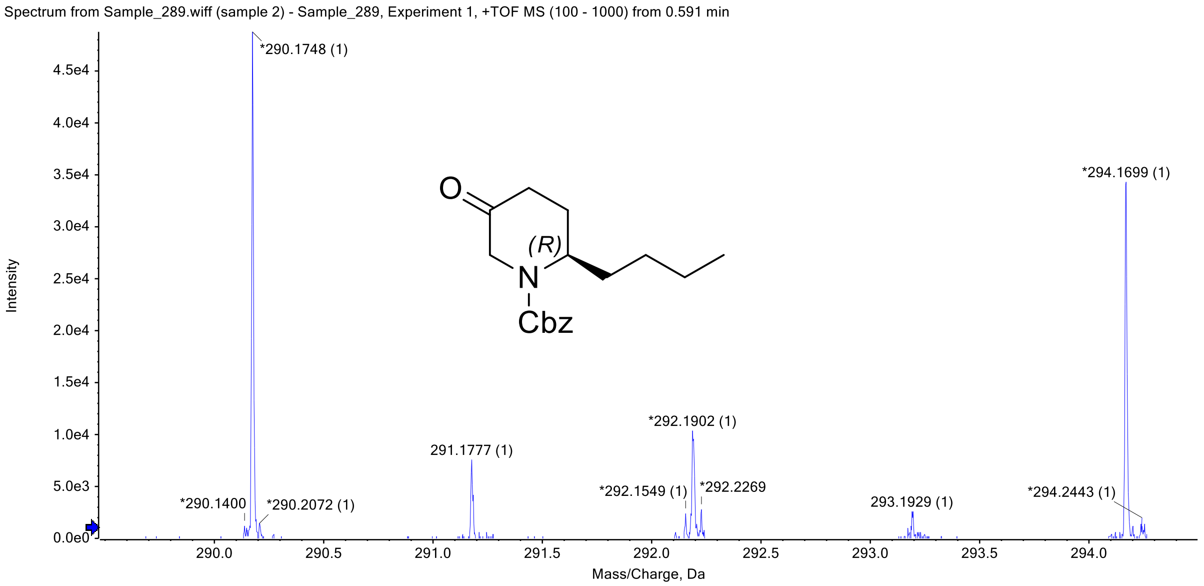

HRMS of compound **9**

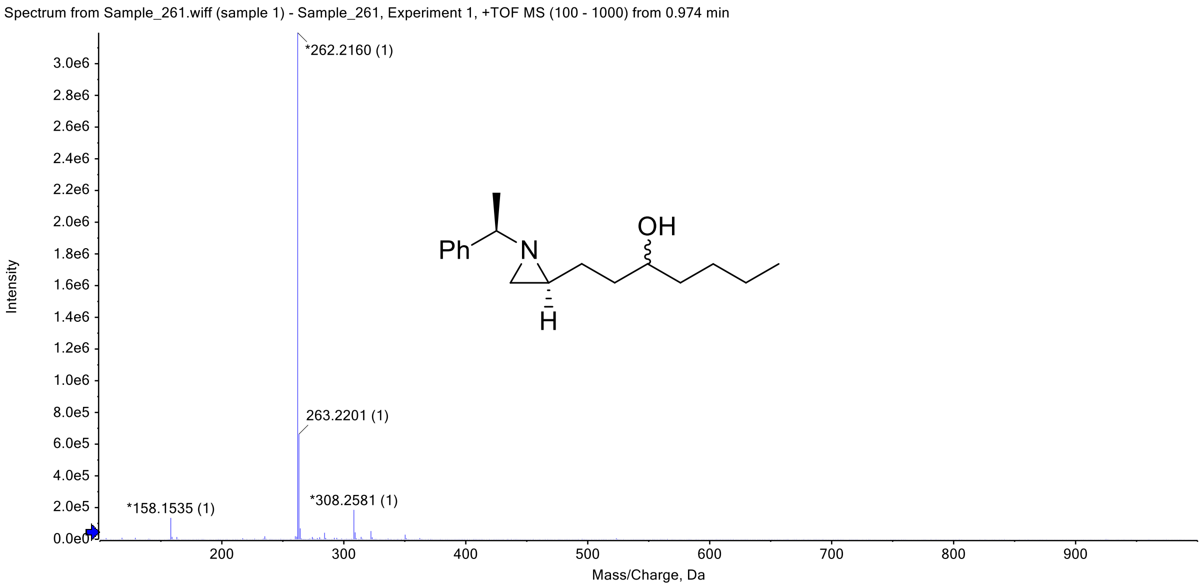

HRMS of compound **10**

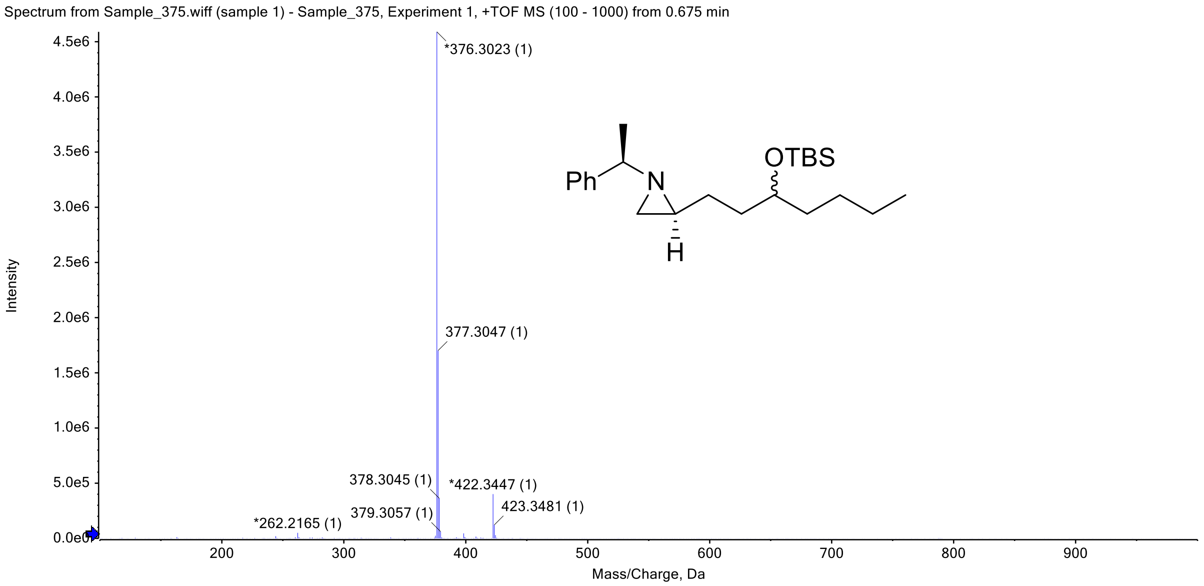

HRMS of compound **11**

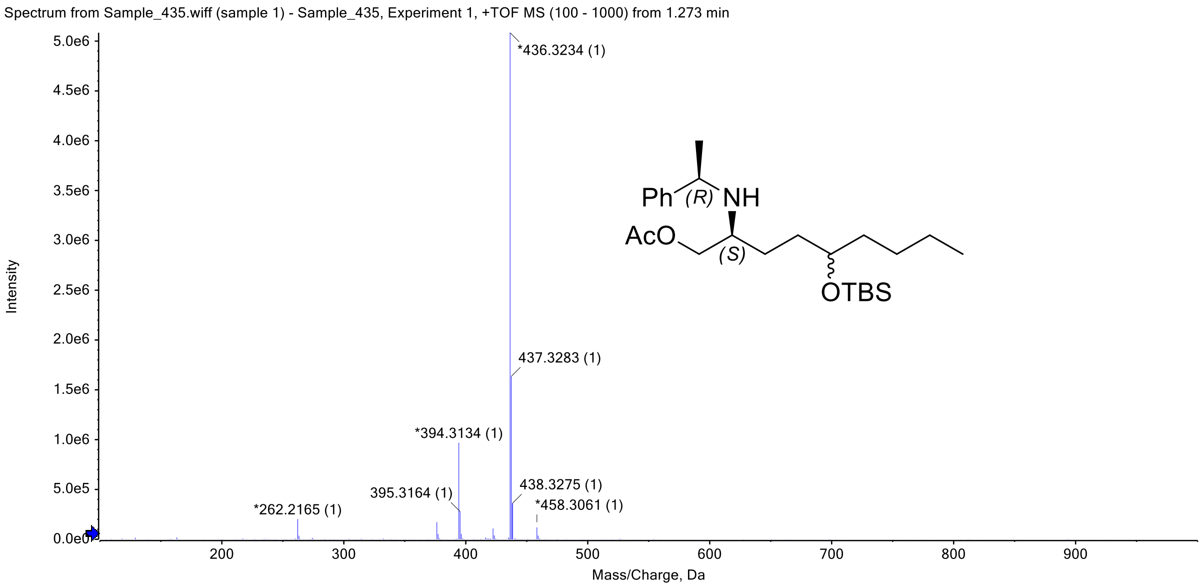

HRMS of compound **12**

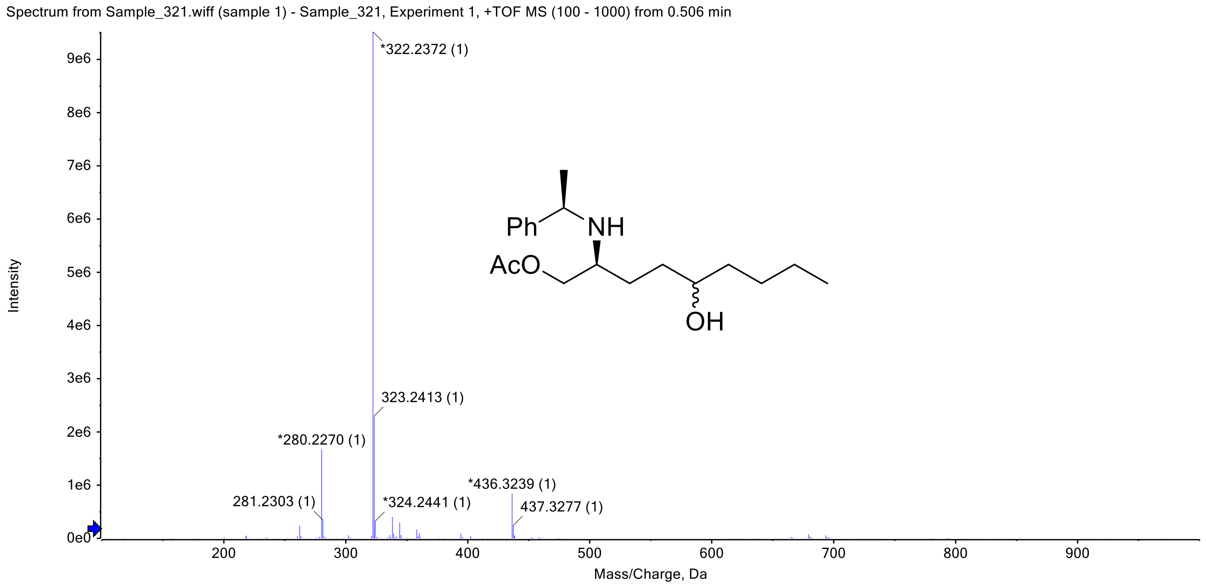

HRMS of compound **13**

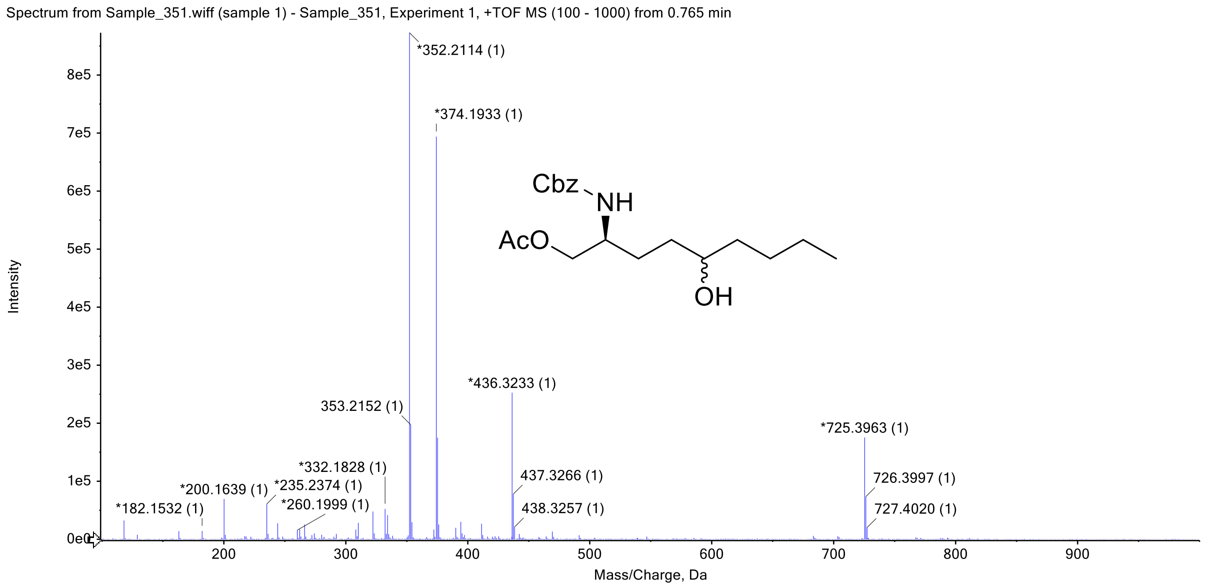

HRMS of compound **14**
